# Systematic review of bovine and zoonotic tuberculosis in the Western Pacific and the Southeast Asia regions of the World Health Organization

**DOI:** 10.3389/fpubh.2024.1345328

**Published:** 2024-07-31

**Authors:** Balbir B. Singh, Navneet K. Dhand, Simeon Cadmus, Anna S. Dean, Corinne S. Merle

**Affiliations:** ^1^Centre for One Health, Guru Angad Dev Veterinary & Animal Sciences University, Ludhiana, India; ^2^One Health Epi Consulting, Glenfield, NSW, Australia; ^3^Sydney School of Veterinary Science, Faculty of Science, The University of Sydney, NSW, Australia; ^4^Centre for Control and Prevention of Zoonoses, University of Ibadan, Ibadan, Nigeria; ^5^Global Tuberculosis Programme, World Health Organization, Geneva, Switzerland; ^6^Special Programme for Research & Training in Tropical Diseases (TDR), World Health Organization, Geneva, Switzerland

**Keywords:** bovine tuberculosis, Southeast Asia, roadmap for zoonotic tuberculosis, Western Pacific, zoonotic tuberculosis

## Abstract

**Introduction:**

Tuberculosis (TB) remains a leading cause of mortality worldwide. We conducted this systematic review to understand the distribution of bovine and zoonotic tuberculosis in the World Health Organization (WHO)’s Southeast Asia Region (SEAR) and Western Pacific Region (WPR) to inform our understanding of the risk posed by this disease.

**Methods:**

A two-pronged strategy was used by evaluating data from peer-reviewed literature and official reports. A systematic search was conducted using a structured query in four databases (Web of Science, Scopus, Medline, and PubMed) to identify any reports of the occurrence of zoonotic TB. No language and time constraints were used during the search, but non-English language articles were later excluded. The official data were sourced from the World Organization for Animal Health’s (WOAH) World Animal Health Information System (WAHIS) and WHO’s global TB database.

**Results:**

The retrieved records from SEAR and WPR (*n* = 113) were screened for eligibility, and data about disease occurrence were extracted and tabulated. In SEAR, all of the five studies that conducted *Mycobacterium* speciation (5/6) in humans were from India, and the reported *Mycobacterium* species included *M. tuberculosis*, *M. bovis*, *M. scrofulacium*, *M. kansasii*, *M. phlei*, *M. smegmatis* and *M. orygis*. In WPR, *Mycobacterium* speciation investigations in humans were conducted in Australia (8), China (2), Japan (2), NewZealand (2) and Malaysia (1), and the reported *Mycobacterium* species included *M. bovis*, *M. africanum* and *M. tuberculosis*. Seven countries in WHO’s SEAR have officially reported the occurrence of *Mycobacterium bovis* in their animals: Bangladesh, India, Indonesia, Myanmar, Nepal, Sri Lanka and Thailand. In WPR, the WAHIS information system includes reports of the identification of *M. bovis* from 11 countries – China, Fiji, Japan, Malaysia, Mongolia, New Zealand, the Philippines, the Republic of Korea, Singapore, Tonga and Viet Nam. In contrast, human zoonotic TB cases in the WHO database were only listed from Australia, Brunei Darussalam and Palau countries.

**Discussion:**

The available data suggests under-reporting of zoonotic TB in the regions. Efforts are required to strengthen zoonotic TB surveillance systems from both animal and human health sides to better understand the impact of zoonotic TB in order to take appropriate action to achieve the goal of ending the TB epidemic.

## 1 Introduction

Tuberculosis (TB) is a global public health emergency and the leading cause of infectious disease-related mortality, after COVID-19 (1). It predominantly affects marginalized populations and is associated with poverty (2). Although the number of TB deaths declined from 2005 to 2019, the COVID-19 pandemic has reversed this trend due to impeded access to TB services (1). In 2021, an estimated 10.6 million people were infected with TB, resulting in 1.6 million deaths (1). In line with the United Nations Sustainable Development Goal of ending the TB epidemic, the WHO developed The End TB strategy to reduce incidence by 80% and deaths due to TB by 90% compared to 2015 levels and eliminate associated socio-economic impacts by 2030 (3). The strategy has been prioritized in WHO’s Southeast Asia and Western Pacific regions (SEAR and WPR), the two most populous WHO regions accounting for more than half of the world’s population, with the development of region-specific strategies (4–7). The global targets are currently off-track as the target to reduce TB incidence by 80% and deaths by 90% compared to 2015 levels by 2030 seems unachievable.

Human TB is primarily caused by *Mycobacterium tuberculosis*, but zoonotic TB caused by *M. bovis* also contributes to the disease burden. Approximately 140,000 people globally (including 43,400 from SEAR and 18,000 from WPR) contracted zoonotic TB in 2019, resulting in about 11,400 (including 2,020 from SEAR and 270 from WPR) deaths (8). Zoonotic TB in people predominantly arises from the transmission of *M. bovis* from cattle, referred to as bovine TB. *M. bovis* not only impacts human health but also causes economic losses and trade barriers, affecting the livelihoods of marginalized communities. Diagnosing and treating zoonotic TB presents challenges because *M. bovis* is naturally resistant to pyrazinamide, a standard first-line TB treatment drug, and routine surveillance systems do not identify zoonotic TB cases. Investigating and acting on zoonotic TB is significant for protecting public health, safeguarding economic stability and ensuring global health security. Zoonotic TB poses a direct threat to public health. Humans can contract the disease through direct contact with infected animals or consumption of contaminated animal products, leading to severe health complications. Secondly, zoonotic TB has substantial economic implications. The disease affects livestock, reducing productivity and causing economic losses for farmers and communities dependent on animal husbandry. Additionally, *M. bovis* is often associated with extra-pulmonary TB. The Survival and ongoing transmission of *M. bovis* in its animal reservoirs is a serious deterrent for The End TB strategy in human populations, in particular when human–livestock interactions are not uncommon in TB-endemic areas.

In 2017, WHO, the World Organization for Animal Health (WOAH), the Food and Agriculture Organization of the United Nations (FAO), and the International Union Against Tuberculosis and Lung Disease (The Union) launched the ‘Roadmap for Zoonotic TB’. This roadmap recommends using the One Health approach to tackle zoonotic TB in animals and humans and identifies ten priorities to reduce disease transmission at the human-animal interface. The reduction of transmission at this interface is one of the three recommended axes for investment outlined by the roadmap. More recently, WHO, FAO, and WOAH published the Tripartite Zoonoses Guide, promoting operational multisectoral tools and one health approach to address zoonotic diseases (9). Additionally, FAO, United Nations Environment Programme (UNEP), WOAH, and WHO (collectively known as the Quadripartite) have developed the One Health Joint Plan of Action (2022–2026) to address challenges at the human-animal–plant-environment interface on regional, national, and global scales (10).

We conducted this systematic review of scientific literature and official databases of WHO and WOAH to understand the current status of bovine and zoonotic TB in WHO’s SEAR and WPR to provide an updated consolidated evidence base on the burden of zoonotic and bovine TB. This information will be used to assess the level of implementation of the Roadmap.

## 2 Methods

We used a two-pronged strategy by evaluating the peer-review literature and data from official reports.

### 2.1 Target countries

**Figure 1:**
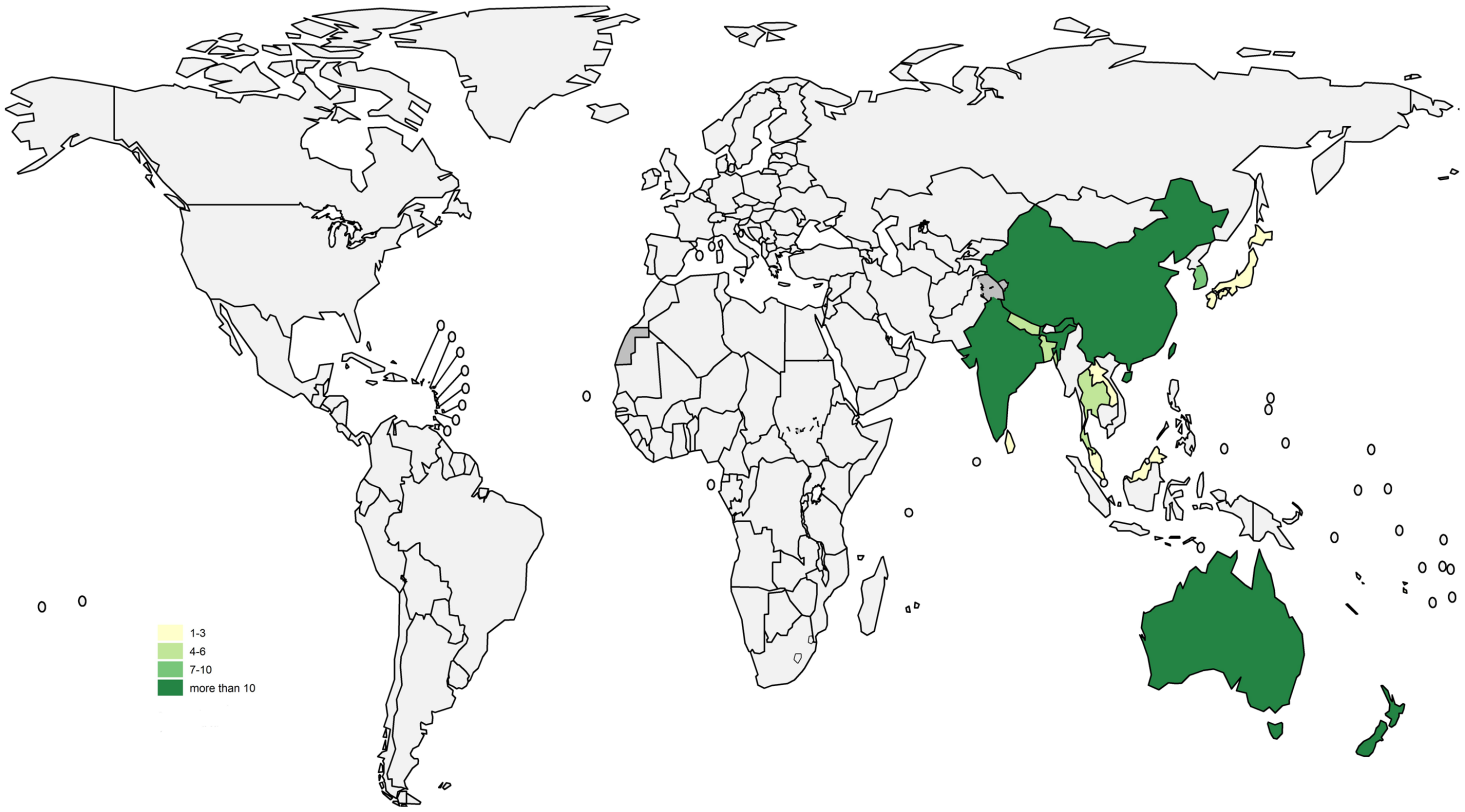
Map showing the number of selected studies in different countries.

We conducted the review for all 11 countries in SEAR: Bangladesh, Bhutan, Democratic People’s Republic of Korea, India, Indonesia, Maldives, Myanmar, Nepal, Sri Lanka, Thailand, and Timor-Leste (11). All 37 countries in WPR were also included. These were: American Samoa, Australia, Brunei Darussalam, Cambodia, China, Cook Islands, Fiji, French Polynesia (France), Guam (USA), Hong Kong SAR (China), Japan, Kiribati, Lao People’s Democratic Republic, Macao SAR (China), Malaysia, Marshall Islands, Micronesia Federated States of, Mongolia, Nauru, New Caledonia (France), New Zealand, Niue, Northern Mariana Islands Commonwealth of the (USA), Palau, Papua New Guinea, Philippines, Pitcairn Island (UK), Republic of Korea, Samoa, Singapore, Solomon Islands, Tokelau (New Zealand), Tonga, Tuvalu, Vanuatu, Viet Nam, Wallis and Futuna (France) (12) (Figure 1).

### 2.2 Search strategy and study selection

Following the PRISMA guidelines (Supplementary Tables S1, S2), we conducted a systematic review to investigate the occurrence of bovine TB and zoonotic TB among animals and humans in the selected countries (13). The search was conducted in August 2022 using four databases – Web of Science, Scopus, Medline, and PubMed. The search strategy described in Box 1 was applied, restricting the specified keywords to the title/abstract of the article. No language and time constraints were used during the search, but non-English language articles were later excluded. The search records were imported to Endnote, duplicate entries were removed, and the abstracts and titles were screened for eligibility using the criteria presented in Table 1. Two reviewers (BBS and ND) independently screened and collected the TB reporting data. In addition, data about the host country, *Mycobacterium* species involved, hosts, and other relevant factors were extracted.

**Table 1:** Eligibility criteria used to select research articles on zoonotic/bovine TB in the target countries of WHO’s Southeast Asia and Western Pacific regions.

Category	Inclusion	Exclusion
Population	Studies reporting the TB infection or disease in vertebrates (domestic or wildlife) and/or zoonotic TB in people in the countries in WHO’s SEAR and WPR.	Studies reporting only *human tuberculosis* (non-zoonotic tuberculosis) in people, infection/disease due to other pathogens or the studies conducted in countries outside the WHO’s SEAR and WPR.
Exposure	Intra dermal skin testing/serological tests/microbiological/molecular confirmation in animals; microbiological/molecular confirmation of zoonotic TB pathogens in people	Other diagnostic tests
Comparator	None	
Outcomes	TB infection or disease in vertebrates; zoonotic tuberculosis in people	Non TB infection or disease in vertebrates; non zoonotic tuberculosis or any other disease in people
Study type	Cross-sectional/case reports/outbreak/systematic review	Experimental or laboratory studies, studies on *M bovis* BCG, diagnostic or vaccination development, disease modeling, control programs, market chain analysis and risk assessment of bovine TB.

**BOX 1 Queries used to search for peer-reviewed articles about the occurrence of zoonotic/bovine TB in the target countries of WHO’s SEAR and WPR.**

**Table:** 

Region	Keywords
Southeast Asia	(Thailand) OR (Bhutan) OR (Indonesia) OR (Democratic People’s Republic of Korea) OR (Sri Lanka) OR (Maldives) OR (Myanmar) OR (Nepal) OR (Bangladesh) OR (Timor-Leste) OR (India) OR (Southeast Asia) OR (SE Asia)
AND	
(*Mycobacterium bovis*) OR (*M bovis*) OR (zTB) OR (zoonotic tuberculosis) OR (*M orygis*) OR (*Mycobacterium orygis*) OR (bovine tuberculosis)	
Western Pacific	(American Samoa) OR (Australia) OR (Brunei Darussalam) OR (Cambodia) OR (China) OR (Cook Islands) OR (Fiji) OR (French Polynesia France) OR (Guam USA) OR (Hong Kong SAR China) OR (Japan) OR (Kiribati) OR (Lao People’s Democratic Republic) OR (Macao SAR China) OR (Malaysia) OR (Marshall Islands) OR (Micronesia Federated States of) OR (Mongolia) OR (Nauru) OR (New Caledonia France) OR (New Zealand) OR (Niue) OR (Northern Mariana Islands Commonwealth of the USA) OR (Palau) OR (Papua New Guinea) OR (Philippines) OR (Pitcairn Island UK) OR (Republic of Korea) OR (Samoa) OR (Singapore) OR (Solomon Islands) OR (Tokelau New Zealand) OR (Tonga) OR (Tuvalu) OR (Vanuatu) OR (Viet Nam) OR (Wallis and Futuna France) OR (Western Pacific)
AND	
(*Mycobacterium bovis*) OR (*M bovis*) OR (zTB) OR (zoonotic tuberculosis) OR (*M orygis*) OR (*Mycobacterium orygis*) OR (bovine tuberculosis)	

### 2.3 Other sources of data

Official animal disease report data were sourced from WOAH’s World Animal Health Information System – WAHIS (14). Data on human cases of zoonotic TB were sourced from WHO’s global TB database. These were only available for 2018 (15).

## 3 Results

### 3.1 Systematic review

**Figure 2:**
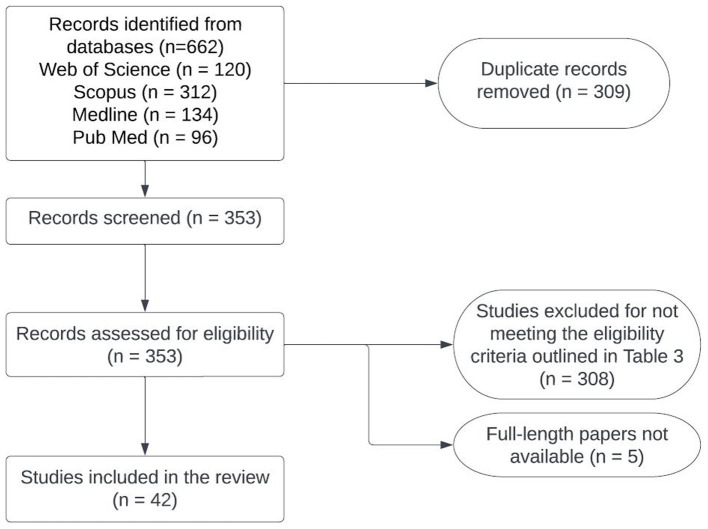
Flowchart of the process used for selecting studies to conduct situation analyses of zoonotic TB in Southeast Asia.

**Figure 3:**
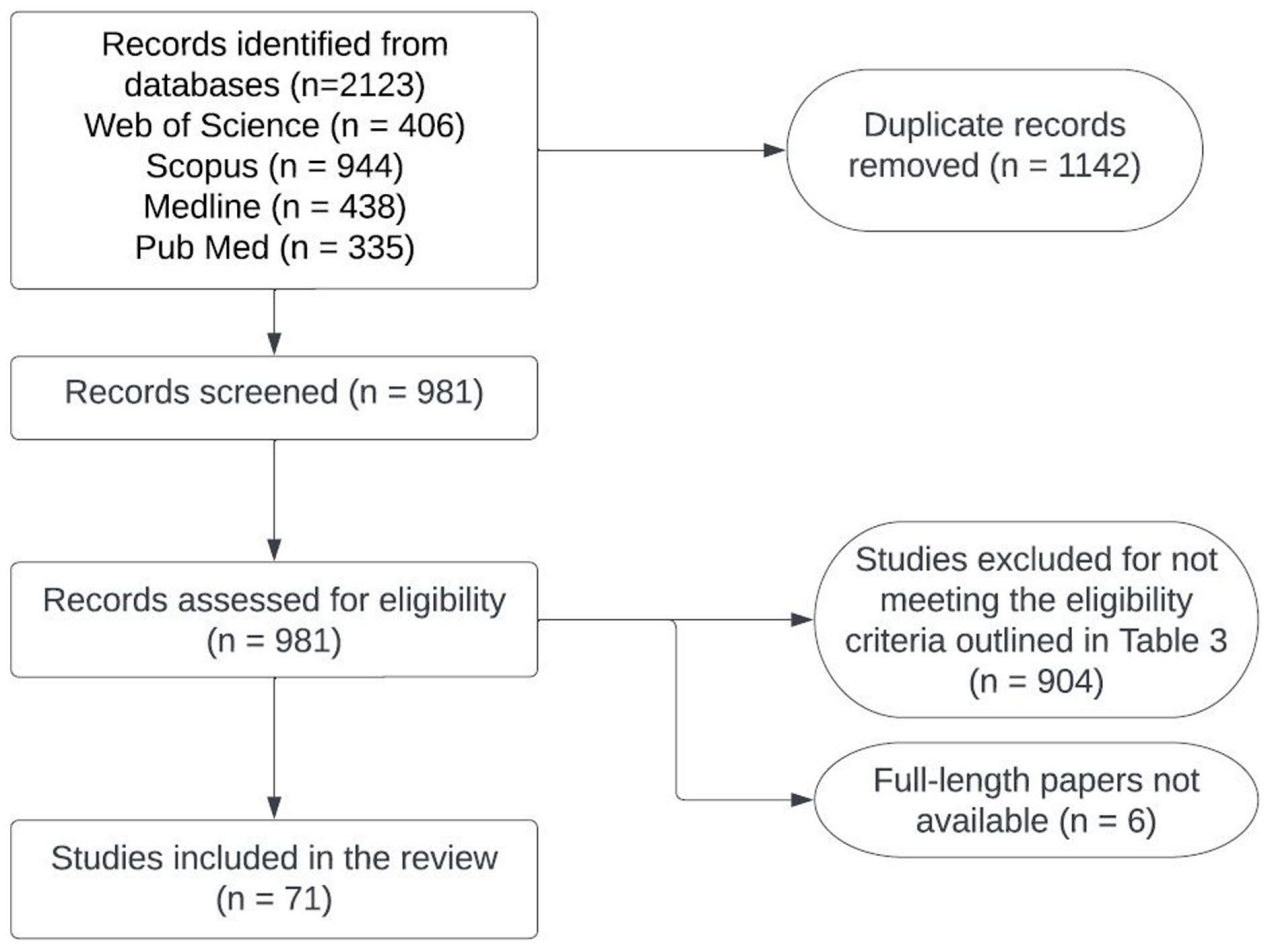
Flowchart of the process used for selecting studies to conduct situation analyses of zoonotic TB in the Western Pacific region.

Detailed information about the number of studies selected at each stage in SEAR and WPR is provided in Figures 2, 3. Data extracted from 113 selected studies conducted in both regions (SEAR = 42; WPR = 71) are presented in Supplementary Tables S3, S4. These studies were conducted in India (25), New Zealand (23), Australia (18), China (12), the Republic of Korea (11), Bangladesh (6), Nepal (5), Thailand (5), Japan (3), Malaysia (2), Fiji (1), Lao PDR (1) and Sri Lanka (1) (Table 2). Overall, limited studies have been conducted to assess zoonotic TB in humans in South East Asia and Western Pacific countries (Supplementary Tables S3, S4). No data on the status of zoonotic TB or bovine TB are available from many South East Asia and Western Pacific region countries.

**Table 2:** Studies conducted on bovine TB or zoonotic TB in different countries in the Southeast Asia and Western Pacific regions.

Country	WHO region	Bovine	Bovine and Wildlife	Bovine, Ovine and Wildlife	Bovine, Swine and Wildlife	Camel	Canine	Caprine	Feline	Humans	Humans and Bovine	Humans and Wildlife	Humans, Canine and wildlife	Swine	Wildlife	Total
Australia	WP	3						1		8		1			5	18
Bangladesh	SEA	5	1													6
China	WP	8								1	1				2	12
Fiji	WP	1														1
India	SEA	15				1				5	1				3	25
Japan	WP	1								1			1			3
Lao PDR	WP	1														1
Malaysia	WP									1					1	2
Nepal	SEA	3													2	5
New Zealand	WP	2	1				1		1	2					16	23
Republic of Korea	WP	4	1	1	1									1	3	11
Sri Lanka	SEA	1														1
Thailand	SEA	3													2	5
Total		47	3	1	1	1	1	1	1	18	2	1	1	1	34	113

#### 3.1.1 Human health

There were 90.9% (20/22) investigations where *Mycobacterium* speciation was conducted in humans – 83.3% (5/6/) in SEAR and 93.7% (15/16) in the WPR. In SEAR, all the five studies that conducted *Mycobacterium* speciation (5/6) in humans were from India, and the reported *Mycobacterium* species included *M. tuberculosis*, *M. bovis*, *M. scrofulacium*, *M. kansasii*, *M. phlei*, *M. smegmatis* and *M. orygis*. In the WPR, the *Mycobacterium* speciation investigations in humans were conducted in Australia (8), China (2), Japan (2), NewZealand (2) and Malaysia (1), and the reported *Mycobacterium* species included *M. bovis*, *M. africanum* and *M. tuberculosis*. *M. bovis*-associated zoonotic TB cases were reported from India. Cases of zoonotic TB have also been reported in Australia, New Zealand and China. *M. orygis* has been reported in human patients in India.

#### 3.1.2 Animal health

There were 64.9% (61/94) investigations where *Mycobacterium* speciation was conducted in animals – 56.7% (21/37) in SEAR and 70.1% (40/57) in the WPR. In SEAR, the pathogens reported from bovines and wildlife included *M. africanum* subtype I (human pathogen), *M. bovis*, *M. orygis*, *M. tuberculosis*, *M. fortuitum*, *M. phlei* and *M. smegmatis* (Supplementary Tables S3, S4). Bovine TB cases associated with *M. orygis* have been reported from Bangladesh, India, and Nepal (Supplementary Table S3). In the studies with speciation data in South East Asia, *M. bovis* TB infections were reported from Bangladesh, India, Nepal, Sri Lanka and Thailand (Supplementary Table S3). In WPR, the pathogens reported from bovine and wildlife included *M. bovis*, *M. orygis*, *M. tuberculosis*, *M. elephantis*, *M. pinnipedii*, and *M. chelonae* (Supplementary Table S4). In the studies with speciation data in WPR, *M. bovis* TB was reported in China, South Korea, and New Zealand. Australia has successfully eradicated bovine TB. A bovine TB case associated with *M. orygis* has been reported from New Zealand (Supplementary Table S4).

*Mycobacterium bovis, M. orygis* and *M. tuberculosis* have been reported from wildlife in SEAR (Supplementary Table S3). *M. bovis* and *M. pinnipedii* have been reported from wildlife in the WPR (Supplementary Table S4).

### 3.2 Results of analysis of official data

The official TB data from WOAH and WHO are summarized in Supplementary Tables S5, S6, and Table 3. Briefly, bovine TB infection in domestic animals in SEAR was reported from Bangladesh, India, Indonesia, Myanmar, Nepal, Sri Lanka and Thailand during 2005–2018 (Supplementary Table S5). In WPR, bovine TB infection among domestic animals was reported in China, Fiji, Japan, Malaysia, Mongolia, New Zealand, the Philippines, the Republic of Korea, Singapore, Tonga and Viet Nam (Supplementary Table S6). The bovine TB infection in wildlife was reported in New Zealand, Mongolia, and Singapore and reported and/or suspected in Fiji, Tonga and Vietnam (Supplementary Table S6). No bovine TB data were available from other countries. For zoonotic TB, *M. bovis* was reported in Australia, Brunei Darussalam and Palau in humans in 2018 (Table 3). Zoonotic TB data were missing from many countries in WPR and SEAR (Supplementary Tables S3, S4, and Table 3).

**Table 3:** WHO compiled zoonotic TB data from the Western Pacific and Southeast regions in 2018 under the global tuberculosis program (https://www.who.int/teams/global-tuberculosis-programme/data).

Country	Zoonotic pulmonary TB		Zoonotic extra-pulmonary TB		
	Number with test results for the speciation of *Mycobacterium tuberculosis* complex (A)	Number with *M. bovis* (B)	Number of bacteriologically confirmed TB cases (C)	Number of TB cases with M tuberculosis (D)	Number of TB cases with *M. bovis* (E)
American Samoa			1	1	0
Australia	810	2	515	515	2
Bangladesh	2	0			
Brunei Darussalam	201	1			
China, Hong Kong SAR	2,734	0	352	352	0
Fiji	143	0	13	13	0
Guam	46	0			
Malaysia	79	0	3,726	22	0
Micronesia	23	0			
Mongolia	1,521	0	184	184	0
New Caledonia	24	0	7	7	0
New Zealand	2	0	2	2	0
Northern Mariana Islands	15	0	3	3	0
Palau	12	12			
Philippines	1,472				
Republic of Korea	1755	0			

The data were only available for 2018 and should therefore be interpreted with caution. A. Among bacteriologically confirmed new and relapse pulmonary TB cases, the number of cases with test results for the speciation of the Mycobacterium tuberculosis complex. B. Among pulmonary cases with test results for speciation reported (B), the number of cases identified as having *Mycobacterium bovis*. C. Among the new and relapse extrapulmonary TB cases reported, the number of bacteriologically confirmed cases, i.e., by culture or molecular technologies. D. Among bacteriologically confirmed extrapulmonary TB cases reported in E, the number of cases with test results for the speciation of the Mycobacterium tuberculosis complex. E. Among extrapulmonary TB cases with test results for speciation reported in F, the number of cases identified as having *Mycobacterium bovis*.

## 4 Discussion

The Asia-Pacific region, comprising the WHO’s SEAR and WPR, is a major hotspot for TB, with a high burden of cases and deaths. These regions accounted for approximately 63% of the estimated 10.6 million global TB cases in 2021 (1). Four countries in WPR, namely Viet Nam, Lao People’s Democratic Republic (PDR), China, and the Philippines, accounted for 90% of new incident cases and 82% of TB-related deaths in 2019 (16). The COVID-19 pandemic has jeopardized the ongoing TB control efforts. To reinvigorate these efforts, the WHO’s SEAR office has developed a new strategic plan for the 2021–2025 (6).

This systematic review aimed to assess the burden of bovine and zoonotic TB in the region and inform the ongoing control efforts, particularly the Roadmap for zoonotic TB launched by the WHO in 2017. The review aimed to identify countries where bovine and zoonotic TB have been reported and countries with no available information on these forms of TB. The review findings indicate that zoonotic TB is not limited to infections caused by *M. bovis*. The role of *M. orygis* in zoonotic TB epidemiology is gaining importance and requires further investigation. *M. orygis* has been reported from human patients in India, and the pathogen has been reported from bovines in Bangladesh, India, and Nepal in South East Asia. A bovine case of *M. orygis* transmitted by an immigrant suffering with *M. orygis* associated TB has also been reported in New Zealand. Currently, the *M. orygis* appears to be circulating in India and its neighboring countries, but its global spread in the near future cannot be ruled out. Differentiating pathogen species in zoonotic TB infections is important for informing TB control efforts. Detecting *M. tuberculosis* infections in cattle and the possibility of human-to-bovine transmission can complicate TB eradication efforts.

The true impact of zoonotic TB in SEAR and WPR remains largely unknown. In 2019, there were an estimated 43,400 zoonotic TB cases and 2020 deaths in Southeast Asia, and 18,000 cases and 270 deaths in Western Pacific (8). Having reliable regional, national, and subnational data is crucial for policy development and determining zoonotic TB control priorities. In countries like Indonesia and Myanmar, among the 30 countries defined by WHO between 2021 and 2025 as high-TB burden (8), there is a lack of data on the presence/absence of bovine TB or on the prevalence of infection in cattle populations despite the fact that there are substantial cattle populations and interactions at the human-bovine interface in these countries. Efforts are needed to estimate the true burden of zoonotic TB in these regions.

The One Health approach, integrating zoonotic TB surveillance and control strategies in humans, bovines, and wildlife, has been advocated to end the zoonotic TB (17). This approach recommends estimating the zoonotic TB burden, using novel diagnostic tools, and implementing One Health interventions to improve zoonotic TB response and prevention (17). The approaches such as the estimation of zDALYs (zoonotic disability adjusted life years), including animal loss equivalents (ALE), have been recommended for zoonotic diseases (18). Developing prompt bovine and zoonotic TB notification capable surveillance systems, roadmaps for enhanced intersectoral coordination, and the use of both WOAH and WHO joint external evaluation tools is recommended. Encouraging milk pasteurization, mandatory meat inspection and condemnation of infected carcasses are important strategies to be adopted in the animal health sector (19). Environment focused strategies such as having dispersed water sources and the use of hedgerows on boundaries could reduce the risk of bTB transmission (19, 20). However, not much progress using such approaches has been achieved in many countries in the Southeast Asia and Western Pacific regions.

The lack of data reported to WHO for 2018 for zoonotic TB in people indicates that these regions need to establish zoonotic TB surveillance systems, strengthen diagnostic laboratory capacity, and incorporate species differentiation into routine TB diagnostic protocols.

The systematic review utilized multiple sources to compile data on bovine and zoonotic TB infections in the Southeast Asia and Western Pacific regions. However, the search strategy focused on country-level keywords, potentially excluding some studies. Additionally, the exclusion of non-English literature may have resulted in missed studies. It is also possible that studies available in national or subnational databases were not included. Nevertheless, the study’s purpose of highlighting the bovine and zoonotic TB situation in the regions was achieved.

## 5 Conclusion

In conclusion, zoonotic TB is not much investigated in SEAR and WPR, emphasizing the need to enhance surveillance and strengthen diagnostic laboratory capacity and capability to achieve the goal of human TB eradication.

## Data availability statement

The original contributions presented in the study are included in the article/Supplementary material, further inquiries can be directed to the corresponding author.

## Author contributions

BS: Formal analysis, Writing – original draft. ND: Formal analysis, Methodology, Writing – review & editing. SC: Methodology, Writing – review & editing. AD: Conceptualization, Funding acquisition, Supervision, Writing – review & editing. CM: Conceptualization, Funding acquisition, Methodology, Supervision, Writing – review & editing.
