# Supplementary material for: Systematic review of bovine and zoonotic tuberculosis in the Western Pacific and the Southeast Asia regions of the World Health Organization
Source: Front Public Health. 2024 Jul 31;12:1345328. doi: 10.3389/fpubh.2024.1345328 (PMC11334259; doi:10.3389/fpubh.2024.1345328)
Supplement: Supplementary file 1 [file Table_1.DOCX]

**Supplementary appendix**

**Table S1.** PRISMA 2020 checklist (Preferred Reporting System for Systematic review and Meta-analysis 2020 checklist): recommended items to address in a systematic review protocol.

| **Section and Topic** | **Item No.** | **Checklist item** | **Location where item is reported** |
| --- | --- | --- | --- |
| **TITLE** | | |  |
| Title | 1 | Identify the report as a systematic review. | 1 |
| **ABSTRACT** | | |  |
| Abstract | 2 | See the PRISMA 2020 for Abstracts checklist. | 1, 2 |
| **INTRODUCTION** | | |  |
| Rationale | 3 | Describe the rationale for the review in the context of existing knowledge. | 3, 4 |
| Objectives | 4 | Provide an explicit statement of the objective(s) or question(s) the review addresses. | 4 |
| **METHODS** | | |  |
| Eligibility criteria | 5 | Specify the inclusion and exclusion criteria for the review and how studies were grouped for the synthesis. | 5 |
| Information sources | 6 | Specify all databases, registers, websites, organizations, reference lists and other sources searched or consulted to identify studies. Specify the date when each source was last searched or consulted. | 5 |
| Search strategy | 7 | Present the full search strategies for all databases, registers and websites, including any filters and limits used. | 5, 6 |
| Selection process | 8 | Specify the methods used to decide whether a study met the inclusion criteria of the review, including how many reviewers screened each record and each report retrieved, whether they worked independently, and if applicable, details of automation tools used in the process. | 5, 6 |
| Data collection process | 9 | Specify the methods used to collect data from reports, including how many reviewers collected data from each report, whether they worked independently, any processes for obtaining or confirming data from study investigators, and if applicable, details of automation tools used in the process. | 5 |
| Data items | 10a | List and define all outcomes for which data were sought. Specify whether all results that were compatible with each outcome domain in each study were sought (e.g. for all measures, time points, analyses), and if not, the methods used to decide which results to collect. | 5 |
|  | 10b | List and define all other variables for which data were sought (e.g. participant and intervention characteristics, funding sources). Describe any assumptions made about any missing or unclear information. | 5 |
| Study risk of bias assessment | 11 | Specify the methods used to assess risk of bias in the included studies, including details of the tool(s) used, how many reviewers assessed each study and whether they worked independently, and if applicable, details of automation tools used in the process. | 5 |
| Effect measures | 12 | Specify for each outcome the effect measure(s) (e.g. risk ratio, mean difference) used in the synthesis or presentation of results. | - |
| Synthesis methods | 13a | Describe the processes used to decide which studies were eligible for each synthesis (e.g. tabulating the study intervention characteristics and comparing against the planned groups for each synthesis | - |
|  | 13b | Describe any methods required to prepare the data for presentation or synthesis, such as handling of missing summary statistics, or data conversions. | - |
|  | 13c | Describe any methods used to tabulate or visually display results of individual studies and syntheses. | - |
|  | 13d | Describe any methods used to synthesize results and provide a rationale for the choice(s). If meta-analysis was performed, describe the model(s), method(s) to identify the presence and extent of statistical heterogeneity, and software package(s) used. | - |
|  | 13e | Describe any methods used to explore possible causes of heterogeneity among study results (e.g. subgroup analysis, meta-regression). | - |
|  | 13f | Describe any sensitivity analyses conducted to assess robustness of the synthesized results. | - |
| Reporting bias assessment | 14 | Describe any methods used to assess risk of bias due to missing results in a synthesis (arising from reporting biases). | - |
| Certainty assessment | 15 | Describe any methods used to assess certainty (or confidence) in the body of evidence for an outcome. | - |
| **RESULTS** | | |  |
| Study selection | 16a | Describe the results of the search and selection process, from the number of records identified in the search to the number of studies included in the review, ideally using a flow diagram. | 7 |
|  | 16b | Cite studies that might appear to meet the inclusion criteria, but which were excluded, and explain why they were excluded. | - |
| Study characteristics | 17 | Cite each included study and present its characteristics. | Supplementary appendix |
| Risk of bias in studies | 18 | Present assessments of risk of bias for each included study. | - |
| Results of individual studies | 19 | For all outcomes, present, for each study: (a) summary statistics for each group (where appropriate) and (b) an effect estimate and its precision (e.g. confidence/credible interval), ideally using structured tables or plots. | Supplementary appendix |
| Results of synthesis | 20a | For each synthesis, briefly summarize the characteristics and risk of bias among contributing studies. | - |
|  | 20b | Present results of all statistical syntheses conducted. If meta-analysis was done, present for each the summary estimate and its precision (e.g. confidence/credible interval) and measures of statistical heterogeneity. If comparing groups, describe the direction of the effect. | - |
|  | 20c | Present results of all investigations of possible causes of heterogeneity among study results. | - |
|  | 20d | Present results of all sensitivity analyses conducted to assess the robustness of the synthesized results. | - |
| Reporting biases | 21 | Present assessments of risk of bias due to missing results (arising from reporting biases) for each synthesis assessed. | - |
| Certainty of evidence | 22 | Present assessments of certainty (or confidence) in the body of evidence for each outcome assessed. | - |
| **DISCUSSION** | | |  |
| Discussion | 23a | Provide a general interpretation of the results in the context of other evidence. | 10 |
|  | 23b | Discuss any limitations of the evidence included in the review. | 11 |
|  | 23c | Discuss any limitations of the review processes used. | 11 |
|  | 23d | Discuss implications of the results for practice, policy, and future research. | 12 |
| **OTHER INFORMATION** | | |  |
| Registration and protocol | 24a | Provide registration information for the review, including register name and registration number, or state that the review was not registered. | - |
|  | 24b | Indicate where the review protocol can be accessed, or state that a protocol was not prepared. | - |
|  | 24c | Describe and explain any amendments to information provided at registration or in the protocol. | - |
| Support | 25 | Describe sources of financial or non-financial support for the review, and the role of the funders or sponsors in the review. | - |
| Competing interests | 26 | Declare any competing interests of review authors. | - |
| Availability of data, code and other materials | 27 | Report which of the following are publicly available and where they can be found: template data collection forms; data extracted from included studies; data used for all analyses; analytic code; any other materials used in the review. | - |

**Table S2.** PRISMA (Preferred Reporting System for Systematic review and Meta-analysis) 2020 for Abstract Checklist.

| **Section and Topic** | **Item No.** | **Checklist item** | **Reported**  **(Yes/No)** |
| --- | --- | --- | --- |
| **TITLE** | | |  |
| Title | 1 | Identify the report as a systematic review. | Yes |
| **BACKGROUND** | | |  |
| Objectives | 2 | Provide an explicit statement of the main objective(s) or question(s) the review addresses. | Yes |
| **METHODS** | | |  |
| Eligibility criteria | 3 | Specify the inclusion and exclusion criteria for the review. | Yes |
| Information sources | 4 | Specify the information sources (e.g. databases, registers) used to identify studies and the date when each was last searched. | Yes |
| Risk of bias | 5 | Specify the methods used to assess risk of bias in the included studies. | - |
| Synthesis of results | 6 | Specify the methods used to present and synthesize results. | Yes |
| **RESULTS** | | |  |
| Included studies | 7 | Give the total number of included studies and participants and summarise relevant characteristics of studies. | Yes |
| Synthesis of results | 8 | Present results for main outcomes, preferably indicating the number of included studies and participants for each. If meta-analysis was done, report the summary estimate and confidence/credible interval. If comparing groups, indicate the direction of the effect (i.e. which group is favoured). | Yes |
| **DISCUSSION** | | | |
| Limitations of evidence | 9 | Provide a brief summary of the limitations of the evidence included in the review (e.g. study risk of bias, inconsistency and imprecision). | Yes |
| Interpretation | 10 | Provide a general interpretation of the results and important implications. | Yes |
| **OTHER** | | | |
| Funding | 11 | Specify the primary source of funding for the review. | No |
| Registration | 12 | Provide the register name and registration number | No |

**Table S3.** Studies on *zoonotic TB* or *bovine TB* conducted in Southeast Asia identified in the systematic review.

| Country | Publication year | Study type | Mycobacterium species | Species identification | Host animal species | Tests conducted | Reference |
| --- | --- | --- | --- | --- | --- | --- | --- |
| Bangladesh | 2007 | Case report | *M. africanum* subtype I (human pathogen) | Yes | Bovine | Spoligo typing performed on DNA extracts | (1) |
|  | 2007 | Cross-sectional | *Mycobacterium* species | No | Bovine | Tuberculin | (2) |
|  | 2017 | Case report | *M. orygis* | Yes | Bovine and Wildlife | Conventional and molecular typing | (3) |
|  | 2020 | Cross-sectional | *Mycobacterium* species | No | Bovine | Tuberculin | (4) |
|  | 2021 | Cross-sectional | *Mycobacterium* species | No | Bovine | Tuberculin | (5) |
|  | 2022 | Cross-sectional | *M. bovis* | Yes | Bovine | PCR | (6) |
| India | 1992 | Cross-sectional | *M. tuberculosis, M. bovis, M. scrofulacium, M. Kansasii, M. phlei and M. smegmatis* | Yes | Humans | Smear, culture and biochemical tests | (7) |
|  | 2004 | Cross-sectional | *Mycobacterium* species | No | Bovine | Tuberculin | (8) |
|  | 2006 | Cross-sectional | *M. fortuitum, M. phlei and M. smegmatis* | Yes | Bovine | PCR | (9) |
|  | 2007 | Cross-sectional | *Mycobacterium* species | No | Bovine | Tuberculin | (10) |
|  | 2008 | Cross-sectional | *M. bovis* and *M. tuberculosis* | Yes | Bovine | Culture and biochemical tests | (11) |
|  | 2010 | Cross-sectional | *Mycobacterium* species | No | Bovine | Tuberculin | (12) |
|  | 2011 | Cross-sectional | *M. bovis* | Yes | Humans | Culture and PCR | (13) |
|  | 2012 | Case report | *M. tuberculosis and M. bovis* | Yes | Bovine | PCR | (14) |
|  | 2014 | Cross-sectional | *M. tuberculosis* | Yes | Bovine | PCR | (15) |
|  | 2015 | Cross-sectional | *Mycobacterium* species | No | Camel | PCR | (16) |
|  | 2015 | Case report | *M. tuberculosis* | Yes | Wildlife | PCR | (17) |
|  | 2016 | Cross-sectional | *Mycobacterium* species | No | Bovine | Tuberculin | (18) |
|  | 2016 | Cross-sectional | *Mycobacterium* species | No | Bovine | Culture | (19) |
|  | 2017 | Prospective cohort | *M. bovis* | Yes | Humans | PCR | (20) |
|  | 2017 | Cross-sectional | *Mycobacterium* species | No | Humans and Bovine | Tuberculin and clinical exam | (21) |
|  | 2017 | Cross-sectional | *M. tuberculosis* and *M. bovis* | Yes | Bovine | Culture and PCR | (22) |
|  | 2018 | Cross-sectional | *M. bovis* | Yes | Bovine | Tuberculin, gamma interferon assay and PCR | (23) |
|  | 2018 | Cross-sectional | *M. tuberculosis* | Yes | Bovine | Culture and PCR | (24) |
|  | 2018 | Systematic review and meta-analysis | *Mycobacterium* species | No | Bovine | NA | (25) |
|  | 2019 | Case report | *M. orygis* | Yes | Bovine | Whole-genome sequencing | (26) |
|  | 2019 | Cross-sectional | *M. tuberculosis* | Yes | Bovine | Culture and PCR | (27) |
|  | 2020 | Cross-sectional | *M. orygis* and *M. bovis* | Yes | Humans | PCR and/or whole-genome sequencing (WGS) | (28) |
|  | 2020 | Cross-sectional | *M. bovis* | Yes | Humans | PCR | (29) |
|  | 2021 | Cross-sectional | *M. bovis* | Yes | Wildlife | PCR | (30) |
|  | 2022 | Case report | *M. orygis* | Yes | Wildlife | PCR, spoligotyping and whole-genome sequencing | (31) |
| Nepal | 2007 | Cross-sectional | *M. bovis* | Yes | Bovine | Culture | (32) |
|  | 2014 | Cross-sectional | *Mycobacterium* species | No | Bovine | Tuberculin | (33) |
|  | 2015 | Case report | *M. orygis* | Yes | Wildlife | Spoligotyping (SIT587) as well as MIRU-VNTR | (34) |
|  | 2016 | Case report | *M. orygis* | Yes | Wildlife | Spoligotyping as well as MIRU-VNTR | (35) |
|  | 2020 | Cross-sectional | *Mycobacterium* species | No | Bovine | Tuberculin, rapid test and ELISA | (36) |
| Sri Lanka | 2021 | Cross-sectional | *M. bovis* | Yes | Bovine | Culture and PCR | (37) |
| Thailand | 1995 | Case report | *M. bovis* | Yes | Bovine | Culture | (38) |
|  | 2010 | Case report | *M. tuberculosis* | Yes | Wildlife | PCR | (39) |
|  | 2020 | Cross-sectional | *Mycobacterium* species | No | Wildlife | IGRA and tuberculin | (40) |
|  | 2022 | Cross-sectional | *Mycobacterium* species | No | Bovine | PCR | (41) |
|  | 2022 | Cross-sectional | *Mycobacterium* species | No | Bovine | Tuberculin and LAMP | (42) |

1. *Among the 100 evaluated endometrial samples, one was AFB smear positive; none was positive by culture, four were positive by histopathology and 13 were positive by PCR. Of the 13 PCR-positive cases, 38.4% were positive for M. tuberculosis, 23.07% for M. bovis, and 38.4% showed co-infection with both species.*
2. *Out of 123 cattle sampled, 12 cattle (9.76%, 95% CI: 5.37-16.76, p < 0.0001) were positive by the tuberculin test, 46 (37.4%, 95% CI: 28.97-46.62, p = 0.007) were tested positive by the rapid test, and 7 (5.7%, 95% CI: 2.52-11.80, p < 0.0001) by ELISA test.*

**Table S4**. Studies on zoonotic TB conducted in the Western Pacific region.

| Country | Publication year | Study type | Mycobacterium species | Species identification | Host species | Tests conducted | Reference |
| --- | --- | --- | --- | --- | --- | --- | --- |
| Australia | 1980 | Cross-sectional | M. bovis | Yes | Bovine | Culture | (43) |
|  | 1981 | Cross-sectional | M. bovis | Yes | Wildlife | Culture | (44) |
|  | 1981 | Case report | M. bovis | Yes | Wildlife | Culture | (45) |
|  | 1989 | Outbreak | M. bovis | Yes | Wildlife | Culture | (46) |
|  | 1992 | Cross-sectional | M. bovis | Yes | Humans | Culture | (47) |
|  | 1993 | Case report | M. bovis | Yes | Humans and Wildlife | Culture | (48) |
|  | 1993 | Case report | M. bovis | Yes | Caprine | Culture | (49) |
|  | 1993 | Case report | MTBC | Yes | Wildlife | REA | (50) |
|  | 1995 | Cross-sectional | M. bovis | Yes | Wildlife | Culture | (51) |
|  | 1999 | Cross-sectional | M. bovis | Yes | Humans | Culture | (52) |
|  | 2002 | Cross-sectional | M. bovis | Yes | Humans | Culture | (53) |
|  | 2003 | Cross-sectional | M. africanum | Yes | Humans | Culture | (54) |
|  | 2007 | Cross-sectional | M. africanum, M. bovis | Yes | Humans | Culture | (55) |
|  | 2008 | Cross-sectional | M. bovis | Yes | Humans | Culture | (56) |
|  | 2009 | Cross-sectional | M. africanum, M. bovis | Yes | Humans | Culture | (57) |
|  | 2010 | Case report | Mycobacterium species | No | Humans | Tuberculin and ELISA | (58) |
|  | 2011 | Report | Disease freedom | No | Bovine | n/a | (59, 60) |
|  |  |  |  |  |  |  |  |
| China | 2006 | Review (Book chapter) | Mycobacteriun species | No | Bovine | n/a | cited in (61) |
|  | 2009 | Cross-sectional | M. bovis and MTBC | Yes | Humans and Bovine | Culture and PCR | (62) |
|  | 2011 | Cross-sectional | Mycobacterium species | No | Bovine | ELISA | (63) |
|  | 2012 | Cross-sectional | M. bovis | Yes | Bovine | Cervical tuberculin (SICCT) test and IFN-γ assay | (64) |
|  | 2013 | Cross-sectional | Mycobacterium species | No | Wildlife | ELISA | (65) |
|  | 2015 | Case reports | M. bovis | Yes | Wildlife | MIRU-VNTR | (66) |
|  | 2017 | Case report | M. elephantis | Yes | Bovine | PCR and sequencing | (67) |
|  | 2019 | Cross-sectional | Mycobacterium species | No | Bovine | Tuberculin | (68) |
|  | 2020 | Case report | M. bovis | Yes | Humans | Culture | (69) |
|  | 2021 | Cross-sectional | M. bovis | Yes | Bovine | SIT, CIT, IFN-γ assay and ELISA | (70) |
|  | 2021 | Systematic review and meta-analysis | Mycobacteriun species | No | Bovine | n/a | (71) |
|  | 2021 | Systematic review and meta-analysis | Mycobacterium species | No | Bovine | n/a | (72) |
|  | 2006 | Review (Book chapter) | Mycobacteriun species | No | Bovine | n/a | (73) |
|  |  |  |  |  |  |  |  |
| Fiji | 2018 | Cross-sectional | Mycobacterial species | No | Bovine | Tuberculin | (74) |
|  |  |  |  |  |  |  |  |
| Japan | 1941 | Case report | M. bovis | Yes | Humans | Culture | (75) |
|  | 2007 | Outbreak | M. tuberculosis | Yes | Humans, Canine and wildlife | n/a | (76) |
|  | 2010 | Report | Disease freedom | No | Bovine | n/a | (77) |
|  |  |  |  |  |  |  |  |
| Lao PDR | 2012 | Cross-sectional | Mycobacteriun species | No | Bovine | ELISA | (78) |
| Malaysia | 2019 | Cross-sectional | M. bovis | Yes | Humans | Sequencing | (79) |
|  | 2021 | Cross-sectional | Mycobacterium tuberculosis complex (MTBC) | No | Wildlife | ELISA bPPD IgG | (80) |
|  |  |  |  |  |  |  |  |
| New Zealand | 1985 | Case report | M. bovis | Yes | Wildlife | Culture | (81) |
|  | 1986 | Case report | M. bovis | Yes | Wildlife | Molecular techniques | (82) |
|  | 1990 | Case report | M. bovis | Yes | Feline | Culture | (83) |
|  | 1994 | Case report | M. bovis | Yes | Wildlife | Unknown | (84) |
|  | 1995 | Cross-sectional | M. bovis | Yes | Wildlife | Culture | (85) |
|  | 1995 | Cross-sectional | M. bovis | Yes | Wildlife | Histopathological examination | (86) |
|  | 1997 | Cross-sectional | M. bovis | Yes | Wildlife | Culture | (87) |
|  | 1998 | Cross-sectional | M. bovis | Yes | Wildlife | Culture | (88) |
|  | 2000 | case report | M. bovis | Yes | Canine | Microbiological and genetic studies | (89) |
|  | 2002 | Case report | M. bovis | Yes | Humans | Unknown | (90) |
|  | 2003 | Cross-sectional | M. bovis | Yes | Wildlife | Culture | (91) |
|  | 2004 | Cross-sectional | M. bovis | Yes | Wildlife | Tuberculin and blood testing | (92) |
|  | 2004 | Outbreak | Mycobacterium species | No | Wildlife | Tuberculin and ELISA | (93) |
|  | 2006 | Cross-sectional | Mycobacterium species | No | Wildlife | Unknown | (94) |
|  | 2006 | cross-sectional | M. bovis | Yes | Wildlife | Culture | (95) |
|  | 2008 | Cross-sectional | M. bovis | Yes | Wildlife | DNA typing by restriction endonuclease analysis, culture | (96) |
|  | 2012 | Case report | M. orygis | Yes | Bovine | Culture | (97) |
|  | 2016 | Cross-sectional | Mycobacteriun species | No | Bovine | Intradermal caudal fold testing (CFT) and parallel gamma-IFN testing | (98) |
|  | 2018 | cross-sectional | M. bovis | Yes | Humans | Restriction endonuclease analysis (REA) | (99) |
|  | 2019 | Case reports | M. pinnipedii | Yes | Wildlife | PCR | (100) |
|  | 2021 | Meta-regression and systematic review | Mycobacteriun species | No | Wildlife | n/a | (101) |
|  | 1985 | Case report | M. bovis | Yes | Wildlife | Culture | (102) |
|  | 1986 | Case report | M. bovis | Yes | Wildlife | Molecular techniques | (103) |
| Republic of Korea | 2010 | Review | Mycobacteriun species | No | Bovine, Ovine and Wildlife | n/a | Cited in (104) |
|  | 2010 | Cross-sectional | Mycobacterium species | No | Bovine | Tuberculin and ELISA | (105) |
|  | 2015 | Case report | M. bovis | Yes | Wildlife | Molecular analysis | (106) |
|  | 2015 | Case report | M. bovis | Yes | Bovine and Wildlife | MIRU-VNTR | (107) |
|  | 2016 | Case report | M. bovis | Yes | Swine | Culture and typing | (108) |
|  | 2017 | Case report | Mycobacterium chelonae subsp. Bovis subsp. nov | Yes | Bovine | Multilocus sequence typing analysis | (109) |
|  | 2017 | Case report | M. bovis | Yes | Bovine | Genomic analysis | (110) |
|  | 2017 | Cross-sectional | M. bovis | Yes | Wildlife | Spoligotyping | (111) |
|  | 2018 | Case report | M. bovis | Yes | Bovine | Culture and typing | (112) |
|  | 2020 | Case report | M. bovis | Yes | Wildlife | PCR and spoligotyping | (113) |
|  | 2021 | Case report | M. bovis | Yes | Bovine, Swine and Wildlife | Whole-genome sequencing | (114) |

MTBC: Mycobacterium tuberculosis complex

**Table S5.** The number of six-monthly reports substantiating the occurrence of bovine TB in domestic and wild animals in countries of the South East Asia region based on WOAH-WAHIS data. (reproduced from WAHIS, 2022).

| **COUNTRY** | **S**  **P**  **E**  **C**  **I**  **E**  **S** | J  A  N | J  U  L | J  A  N | J  U  L | J  A  N | J  U  L | J  A  N | J  U  L | J  A  N | J  U  L | J  A  N | J  U  L | J  A  N | J  U  L | J  A  N | J  U  L | J  A  N | J  U  L | J  A  N | J  U  L | J  A  N | J  U  L | J  A  N | J  U  L | J  A  N | J  U  L | J  A  N | J  U  L | N | P | S | A |
| --- | --- | --- | --- | --- | --- | --- | --- | --- | --- | --- | --- | --- | --- | --- | --- | --- | --- | --- | --- | --- | --- | --- | --- | --- | --- | --- | --- | --- | --- | --- | --- | --- | --- |
|  |  |  |  |  |  |  |  |  |  |  |  |  |  |  |  |  |  |  |  |  |  |  |  |  |  |  |  |  |  |  |  |  |  |
|  |  | J  U  N | D  E  C | J  U  N | D  E  C | J  U  N | D  E  C | J  U  N | D  E  C | J  U  N | D  E  C | J  U  N | D  E  C | J  U  N | D  E  C | J  U  N | D  E  C | J  U  N | D  E  C | J  U  N | D  E  C | J  U  N | D  E  C | J  U  N | D  E  C | J  U  N | D  E  C | J  U  N | D  E  C |  |  |  |  |
|  |  |  |  |  |  |  |  |  |  |  |  |  |  |  |  |  |  |  |  |  |  |  |  |  |  |  |  |  |  |  |  |  |  |
|  |  | 2005 | | 2006 | | 2007 | | 2008 | | 2009 | | 2010 | | 2011 | | 2012 | | 2013 | | 2014 | | 2015 | | 2016 | | 2017 | | 2018 | |  |  |  |  |
| **BANGLADESH** | D | - | - | - | - |  |  |  |  |  |  |  |  |  |  |  |  |  |  |  |  |  |  |  |  |  |  |  |  | 4 | 24 | 0 | 0 |
|  | W | - | - | - | - |  |  |  |  |  |  |  |  | - | - | - | - | - | - | - | - | - | - | - | - | - | - | - | - | 20 | 8 | 0 | 0 |
| **BHUTAN** | D | - | - | - | - | - | - | - | - |  |  |  |  |  |  |  |  |  |  |  |  |  |  |  |  |  |  |  |  | 8 | 0 | 0 | 20 |
|  | W | - | - | - | - | - | - | - | - |  |  |  |  |  |  |  |  |  |  |  |  |  |  |  |  |  |  |  |  | 8 | 0 | 0 | 20 |
| **DEMOCRATIC PEOPLE'S REPUBLIC OF KOREA** | D | - | - | - | - |  |  | - | - | - | - | - | - | - | - |  |  |  | - |  |  | - | - |  |  | - | - | - | - | 0 | 19 | 0 | 9 |
|  | W | - | - | - | - |  |  | - | - | - | - | - | - | - | - | - | - | - | - | - | - | - | - |  |  | - | - | - | - | 24 | 0 | 0 | 4 |
| **INDIA** | D |  |  |  |  |  |  |  |  |  |  |  |  |  |  |  |  |  | - | - | - | - | - | - | - | - | - | - | - | 11 | 15 | 0 | 2 |
|  | W |  |  |  |  |  |  |  |  | - | - | - | - | - | - | - | - | - | - | - | - | - | - | - | - | - |  | - | - | 19 | 9 | 0 | 0 |
| **INDONESIA** | D |  |  |  |  | - | - |  |  | - | - | - | - | - | - | - | - | - | - | - | - |  |  |  |  |  |  |  |  | 14 | 4 | 0 | 10 |
|  | W |  |  |  |  | - | - |  |  | - | - | - | - | - | - | - | - | - | - | - | - | - | - | - | - | - | - | - | - | 22 | 4 | 0 | 2 |
| **MALDIVES** | D |  |  |  |  |  |  |  |  |  |  |  |  |  |  |  |  |  |  |  |  |  |  |  |  |  |  |  |  | 0 | 0 | 0 | 28 |
|  | W |  |  |  |  |  |  |  |  |  |  |  |  |  |  |  |  |  |  |  |  |  |  |  |  |  |  |  |  | 0 | 0 | 0 | 28 |
| **MYANMAR** | D |  |  |  |  |  |  |  |  |  |  |  |  |  |  |  |  |  |  |  |  |  |  |  |  |  |  |  |  | 0 | 6 | 0 | 22 |
|  | W |  |  |  |  |  |  |  |  |  |  |  | - |  |  |  |  |  |  |  |  |  |  |  |  |  |  |  |  | 1 | 13 | 0 | 14 |
|  |  | 2005 | | 2006 | | 2007 | | 2008 | | 2009 | | 2010 | | 2011 | | 2012 | | 2013 | | 2014 | | 2015 | | 2016 | | 2017 | | 2018 | | N | P | S | A |
| **NEPAL** | D |  |  |  |  |  |  |  |  |  |  |  |  |  |  |  |  |  |  |  |  |  |  |  |  |  |  |  |  | 0 | 0 | 0 | 28 |
|  | W |  |  |  |  |  |  |  |  |  |  |  |  | - | - | - | - | - | - | - | - | - | - | - | - | - | - | - | - | 16 | 4 | 0 | 8 |
| **SRI LANKA** | D |  |  |  |  |  |  |  |  |  |  |  |  |  |  |  |  |  |  |  |  |  |  |  |  |  |  |  |  | 0 | 12 | 0 | 16 |
|  | W |  |  |  |  |  |  |  |  |  |  |  |  |  |  |  |  | - |  |  |  |  |  |  |  |  |  |  |  | 1 | 0 | 0 | 27 |
| **THAILAND** | D |  |  |  |  |  |  |  |  |  |  |  |  |  |  |  |  |  |  |  |  |  |  |  |  |  |  |  |  | 0 | 18 | 0 | 10 |
|  | W |  |  |  |  |  |  |  |  |  |  |  |  |  |  |  |  |  |  |  |  |  |  |  |  |  |  |  |  | 0 | 0 | 0 | 28 |
| **TIMOR-LESTE** | D | - | - | - | - | - | - | - | - | - | - | - | - | - | - | - | - | - | - | - | - | - | - |  |  |  |  |  |  | 22 | 0 | 0 | 6 |
|  | W | - | - | - | - | - | - | - | - | - | - | - | - | - | - | - | - | - | - | - | - | - | - |  |  |  |  |  |  | 22 | 0 | 0 | 6 |

D = Domestic; W = Wildlife

| No information (N) | Present (P) | Suspected (S) | Absent (A) |
| --- | --- | --- | --- |
| - |  |  |  |

**Table S6.** The number of six-monthly reports substantiating the occurrence of bovine TB in domestic and wild animals in countries of the Western Pacific region based on WOAH-WAHIS data. (reproduced from WAHIS, 2022).

| **COUNTRY** | **S**  **P**  **E**  **C**  **I**  **E**  **S** | J  A  N | J  U  L | J  A  N | J  U  L | J  A  N | J  U  L | J  A  N | J  U  L | J  A  N | J  U  L | J  A  N | J  U  L | J  A  N | J  U  L | J  A  N | J  U  L | J  A  N | J  U  L | J  A  N | J  U  L | J  A  N | J  U  L | J  A  N | J  U  L | J  A  N | J  U  L | J  A  N | J  U  L | N | P | S | A |
| --- | --- | --- | --- | --- | --- | --- | --- | --- | --- | --- | --- | --- | --- | --- | --- | --- | --- | --- | --- | --- | --- | --- | --- | --- | --- | --- | --- | --- | --- | --- | --- | --- | --- |
|  |  |  |  |  |  |  |  |  |  |  |  |  |  |  |  |  |  |  |  |  |  |  |  |  |  |  |  |  |  |  |  |  |  |
|  |  | J  U  N | D  E  C | J  U  N | D  E  C | J  U  N | D  E  C | J  U  N | D  E  C | J  U  N | D  E  C | J  U  N | D  E  C | J  U  N | D  E  C | J  U  N | D  E  C | J  U  N | D  E  C | J  U  N | D  E  C | J  U  N | D  E  C | J  U  N | D  E  C | J  U  N | D  E  C | J  U  N | D  E  C |  |  |  |  |
|  |  |  |  |  |  |  |  |  |  |  |  |  |  |  |  |  |  |  |  |  |  |  |  |  |  |  |  |  |  |  |  |  |  |
|  |  | 2005 | | 2006 | | 2007 | | 2008 | | 2009 | | 2010 | | 2011 | | 2012 | | 2013 | | 2014 | | 2015 | | 2016 | | 2017 | | 2018 | |  |  |  |  |
| **AMERICAN SAMOA** | D | - | - | - | - | - | - | - | - | - | - | - | - | - | - | - | - | - | - | - | - | - | - | - | - | - | - | - | - | 28 | 0 | 0 | 0 |
|  | W | - | - | - | - | - | - | - | - | - | - | - | - | - | - | - | - | - | - | - | - | - | - | - | - | - | - | - | - | 28 | 0 | 0 | 0 |
| **AUSTRALIA** | D |  |  |  |  |  |  |  |  |  |  |  |  |  |  |  |  |  |  |  |  |  |  |  |  |  |  |  |  | 0 | 0 | 0 | 28 |
|  | W |  |  |  |  |  |  |  |  |  |  |  |  |  |  |  |  |  |  |  |  |  |  |  |  |  |  |  |  | 0 | 0 | 0 | 28 |
| **BRUNEI DARUSSALAM** | D |  |  |  |  |  |  |  |  |  |  |  |  |  |  |  |  |  |  |  |  |  |  |  |  |  |  |  |  | 0 | 0 | 0 | 28 |
|  | W |  |  |  |  |  |  |  |  |  |  |  |  |  |  |  |  |  |  |  |  |  |  |  |  |  |  |  |  | 0 | 0 | 0 | 28 |
| **CAMBODIA** | D |  |  |  |  |  |  |  |  |  |  |  |  |  |  |  |  |  |  |  |  |  |  |  |  |  |  |  |  | 0 | 0 | 0 | 28 |
|  | W |  |  |  |  |  |  |  |  | - | - | - | - | - | - | - | - | - | - | - | - | - | - | - | - | - | - | - | - | 20 | 0 | 0 | 8 |
| **CHINA** | D |  |  |  |  |  |  |  |  |  |  |  |  |  |  |  |  |  |  |  |  |  |  |  |  |  |  |  |  | 0 | 28 | 0 | 0 |
|  | W |  |  |  |  |  |  |  |  | - | - | - | - | - |  |  |  | - | - | - | - | - | - | - | - | - | - | - | - | 17 | 0 | 0 | 11 |
| **COOK ISLANDS** | D | - | - | - | - | - | - | - | - | - | - | - | - | - | - | - | - | - |  |  |  |  |  |  |  |  |  |  |  | 17 | 0 | 0 | 11 |
|  | W | - | - | - | - | - | - | - | - | - | - | - | - | - | - | - | - | - |  |  |  |  |  |  |  |  |  |  |  | 17 | 0 | 0 | 11 |
| **FIJI** | D | - | - | - | - |  |  |  |  |  |  |  |  |  |  |  |  |  |  |  |  |  |  |  |  |  |  |  |  | 4 | 24 | 0 | 0 |
|  | W | - | - | - | - |  |  |  |  | - | - | - | - | - |  |  |  | - | - |  |  |  |  |  |  |  |  |  |  | 11 | 6 | 11 | 0 |
|  |  | 2005 | | 2006 | | 2007 | | 2008 | | 2009 | | 2010 | | 2011 | | 2012 | | 2013 | | 2014 | | 2015 | | 2016 | | 2017 | | 2018 | | N | P | S | A |
| **FRENCH POLYNESIA (FRANCE)** | D |  |  |  |  |  |  |  |  |  |  |  |  |  |  |  |  |  |  |  |  |  |  |  |  |  |  |  |  | 0 | 0 | 0 | 28 |
|  | W |  |  |  |  |  |  |  |  |  |  |  |  |  |  |  |  |  |  |  |  |  |  |  |  |  |  |  |  | 0 | 0 | 0 | 28 |
| **GUAM (USA)** | D | - | - | - | - | - | - | - | - | - | - | - | - | - | - | - | - | - | - | - | - | - | - | - | - | - | - | - | - | 28 | 0 | 0 | 0 |
|  | W | - | - | - | - | - | - | - | - | - | - | - | - | - | - | - | - | - | - | - | - | - | - | - | - | - | - | - | - | 28 | 0 | 0 | 0 |
| **HONG KONG SAR (CHINA)** | D |  |  |  |  |  |  |  |  |  |  |  |  |  |  |  |  |  |  |  |  |  |  |  |  |  |  |  |  | 0 | 0 | 0 | 28 |
|  | W |  |  |  |  |  |  |  |  | - | - | - | - | - | - | - | - | - | - | - | - | - | - | - | - | - | - | - | - | 20 | 0 | 0 | 8 |
| **JAPAN** | D |  |  |  |  |  |  |  |  |  |  |  |  |  |  |  |  |  |  |  |  |  |  |  |  |  |  |  |  | 0 | 3 | 0 | 25 |
|  | W |  |  |  |  |  |  |  |  |  |  |  |  |  |  |  |  |  |  |  |  |  |  |  |  |  |  |  |  | 0 | 0 | 0 | 28 |
| **KIRIBATI** | D | - | - | - | - | - | - |  |  | - | - |  |  |  |  |  |  |  |  |  |  |  |  |  |  |  |  |  |  | 8 | 0 | 0 | 20 |
|  | W | - | - | - | - | - | - |  |  | - | - |  |  |  |  |  |  |  |  |  |  |  |  |  |  |  |  |  |  | 8 | 0 | 0 | 20 |
| **LAO PEOPLE'S DEMOCRATIC REPUBLIC** | D |  |  | - | - | - | - |  |  | - | - | - | - | - | - |  |  |  |  |  |  |  |  |  |  |  |  |  |  | 10 | 0 | 0 | 18 |
|  | W |  |  | - | - | - | - |  |  | - | - | - | - | - | - |  |  |  |  |  |  |  |  |  |  |  |  |  |  | 10 | 0 | 0 | 18 |
| **MACAO SAR (CHINA)** | D | - | - | - | - | - | - | - | - | - | - | - | - | - | - | - | - | - | - | - | - | - | - | - | - | - | - | - | - | 28 | 0 | 0 | 0 |
|  | W | - | - | - | - | - | - | - | - | - | - | - | - | - | - | - | - | - | - | - | - | - | - | - | - | - | - | - | - | 28 | 0 | 0 | 0 |
| **MALAYSIA** | D |  |  |  |  |  |  |  |  |  |  |  |  |  |  |  |  |  |  |  |  |  |  |  |  |  |  |  |  | 0 | 12 | 0 | 16 |
|  | W |  |  |  |  |  |  |  |  |  |  |  |  |  |  |  |  |  |  |  |  |  |  |  |  |  |  |  |  | 0 | 0 | 0 | 28 |
| **MARSHALL ISLANDS** | D | - | - | - | - | - | - | - | - | - | - | - | - | - | - |  |  |  |  |  |  |  |  |  |  |  |  |  |  | 14 | 0 | 0 | 14 |
|  | W | - | - | - | - | - | - | - | - | - | - | - | - | - | - |  |  |  |  |  |  |  |  |  |  |  |  |  |  | 14 | 0 | 0 | 14 |
| **MICRONESIA FEDERATED STATES OF** | D | - | - | - | - | - | - |  |  |  |  |  |  |  |  |  |  |  |  |  |  |  |  |  |  |  |  |  |  | 6 | 0 | 0 | 22 |
|  | W | - | - | - | - | - | - |  |  |  |  |  |  |  |  |  |  |  |  |  |  |  |  |  |  |  |  |  |  | 6 | 0 | 0 | 22 |
|  |  | 2005 | | 2006 | | 2007 | | 2008 | | 2009 | | 2010 | | 2011 | | 2012 | | 2013 | | 2014 | | 2015 | | 2016 | | 2017 | | 2018 | | N | P | S | A |
| **MONGOLIA** | D |  |  |  |  |  |  |  |  |  |  |  |  |  |  |  |  |  |  |  |  |  |  |  |  |  |  |  |  | 0 | 13 | 0 | 15 |
|  | W |  |  |  |  |  |  |  |  | - | - | - | - | - | - | - | - |  |  |  |  |  |  |  |  |  |  |  |  | 8 | 5 | 0 | 15 |
| **NAURU** | D | - | - | - | - | - | - | - | - | - | - | - | - | - | - | - | - | - | - | - | - | - | - | - | - | - | - | - | - | 28 | 0 | 0 | 0 |
|  | W | - | - | - | - | - | - | - | - | - | - | - | - | - | - | - | - | - | - | - | - | - | - | - | - | - | - | - | - | 28 | 0 | 0 | 0 |
| **NEW CALEDONIA (FRANCE)** | D |  |  |  |  |  |  |  |  |  |  |  |  |  |  |  |  |  |  |  |  |  |  |  |  |  |  |  |  | 0 | 0 | 0 | 28 |
|  | W |  |  |  |  |  |  |  |  |  |  |  |  |  |  |  |  |  |  |  |  |  |  |  |  |  |  |  |  | 0 | 0 | 0 | 28 |
| **NEW ZEALAND** | D |  |  |  |  |  |  |  |  |  |  |  |  |  |  |  |  |  |  |  |  |  |  |  |  |  |  |  |  | 0 | 28 | 0 | 0 |
|  | W |  |  |  |  |  |  |  |  |  |  |  |  |  |  |  |  |  |  |  |  |  |  |  |  |  |  |  |  | 0 | 20 | 0 | 8 |
| **NIUE** | D | - | - | - | - | - | - | - | - | - | - | - | - | - | - | - | - | - | - | - | - | - | - | - | - | - | - | - | - | 28 | 0 | 0 | 0 |
|  | W | - | - | - | - | - | - | - | - | - | - | - | - | - | - | - | - | - | - | - | - | - | - | - | - | - | - | - | - | 28 | 0 | 0 | 0 |
| **NORTHERN MARIANA ISLANDS COMMONWEALTH OF THE (USA)** | D | - | - | - | - | - | - | - | - | - | - | - | - | - | - | - | - | - | - | - | - | - | - | - | - | - | - | - | - | 28 | 0 | 0 | 0 |
|  | W | - | - | - | - | - | - | - | - | - | - | - | - | - | - | - | - | - | - | - | - | - | - | - | - | - | - | - | - | 28 | 0 | 0 | 0 |
| **PALAU** | D | - | - | - | - | - | - | - | - | - | - | - | - | - | - |  |  |  |  |  |  |  |  |  |  |  |  |  |  | 14 | 0 | 0 | 14 |
|  | W | - | - | - | - | - | - | - | - | - | - | - | - | - | - |  |  |  |  |  |  |  |  |  |  |  |  |  |  | 14 | 0 | 0 | 14 |
| **PAPUA NEW GUINEA** | D | - | - | - | - | - | - |  |  |  |  |  |  |  |  |  |  |  |  |  |  |  |  |  |  |  |  |  |  | 6 | 0 | 0 | 22 |
|  | W | - | - | - | - | - | - |  |  |  |  |  |  |  |  |  |  |  |  |  |  |  |  |  |  |  |  |  |  | 6 | 0 | 0 | 22 |
| **PHILIPPINES** | D | - | - | - | - | - | - |  |  |  |  |  |  |  |  |  |  |  |  |  |  |  |  |  |  |  |  |  |  | 6 | 0 | 2 | 20 |
|  | W | - | - | - | - | - | - |  |  |  |  |  |  |  |  |  |  |  |  |  |  |  | - | - | - | - | - | - | - | 6 | 0 | 0 | 22 |
| **PITCAIRN ISLAND (UK)** | D | - | - | - | - | - | - | - | - | - | - | - | - | - | - | - | - | - | - | - | - | - | - | - | - | - | - | - | - | 28 | 0 | 0 | 0 |
|  | W | - | - | - | - | - | - | - | - | - | - | - | - | - | - | - | - | - | - | - | - | - | - | - | - | - | - | - | - | 28 | 0 | 0 | 0 |
| **REPUBLIC OF KOREA** | D |  |  |  |  |  |  |  |  |  |  |  |  |  |  |  |  |  |  |  |  |  |  |  |  |  |  |  |  | 0 | 28 | 0 | 0 |
|  | W |  |  |  |  |  |  |  |  |  |  |  |  |  |  |  |  |  |  |  |  |  |  |  |  |  |  |  |  | 0 | 0 | 0 | 28 |
|  |  | 2005 | | 2006 | | 2007 | | 2008 | | 2009 | | 2010 | | 2011 | | 2012 | | 2013 | | 2014 | | 2015 | | 2016 | | 2017 | | 2018 | | N | P | S | A |
| **SAMOA** | D |  |  |  |  |  |  |  |  |  |  |  |  |  |  |  |  |  |  |  |  |  |  |  |  |  |  |  |  | 0 | 0 | 0 | 28 |
|  | W |  |  |  |  |  |  |  |  | - | - | - | - | - | - | - | - | - | - | - | - | - | - | - | - | - | - | - | - | 20 | 0 | 0 | 8 |
| **SINGAPORE** | D |  |  |  |  |  |  |  |  |  |  |  |  |  |  |  |  |  |  |  |  |  |  |  |  |  |  |  |  | 0 | 0 | 0 | 28 |
|  | W |  |  |  |  |  |  |  |  |  |  |  |  |  |  |  |  |  |  |  |  |  |  |  |  |  |  |  |  | 0 | 1 | 0 | 27 |
| **SOLOMON ISLANDS** | D | - | - | - | - | - | - | - | - | - | - | - | - | - | - | - | - | - | - | - | - | - | - | - | - | - | - | - | - | 28 | 0 | 0 | 0 |
|  | W | - | - | - | - | - | - | - | - | - | - | - | - | - | - | - | - | - | - | - | - | - | - | - | - | - | - | - | - | 28 | 0 | 0 | 0 |
| **TOKELAU (NEW ZEALAND)** | D | - | - | - | - | - | - | - | - | - | - | - | - | - | - | - | - | - | - | - | - | - | - | - | - | - | - | - | - | 28 | 0 | 0 | 0 |
|  | W | - | - | - | - | - | - | - | - | - | - | - | - | - | - | - | - | - | - | - | - | - | - | - | - | - | - | - | - | 28 | 0 | 0 | 0 |
| **TONGA** | D | - | - | - | - | - | - |  |  |  |  |  | - |  |  | - | - | - | - | - | - | - | - | - | - |  |  |  |  | 17 | 0 | 7 | 4 |
|  | W | - | - | - | - | - | - |  |  | - | - | - | - | - | - | - | - | - | - | - | - | - | - | - | - | - | - | - | - | 26 | 0 | 2 | 0 |
| **TUVALU** | D | - | - | - | - | - | - | - | - | - | - | - | - | - | - | - | - | - | - | - | - | - | - | - | - | - | - | - | - | 28 | 0 | 0 | 0 |
|  | W | - | - | - | - | - | - | - | - | - | - | - | - | - | - | - | - | - | - | - | - | - | - | - | - | - | - | - | - | 28 | 0 | 0 | 0 |
| **VANUATU** | D |  |  |  |  |  |  |  |  |  |  |  |  |  |  |  |  |  |  |  |  |  |  |  |  |  |  |  |  | 0 | 0 | 0 | 28 |
|  | W |  |  |  |  |  |  |  |  | - | - | - | - | - | - | - | - | - | - | - | - | - | - | - | - | - | - | - | - | 20 | 0 | 0 | 8 |
| **VIET NAM** | D |  |  |  |  |  |  |  |  |  |  |  |  |  |  |  |  |  |  |  |  |  |  |  |  |  |  |  |  | 0 | 0 | 18 | 10 |
|  | W |  |  |  |  |  |  |  |  | - | - | - | - | - | - | - | - | - | - | - | - | - | - | - | - | - | - | - | - | 20 | 0 | 8 | 0 |
| **WALLIS AND FUTUNA (FRANCE)** |  | - | - | - | - |  |  | - | - | - | - | - | - | - | - | - | - | - | - | - | - | - | - | - | - | - | - | - | - | 26 | 0 | 0 | 2 |
|  |  | - | - | - | - |  |  | - | - | - | - | - | - | - | - | - | - | - | - | - | - | - | - | - | - | - | - | - | - | 26 | 0 | 0 | 2 |

D = Domestic; W = Wildlife

| No information (N) | Present (P) | Suspected (S) | Absent (A) |
| --- | --- | --- | --- |
| - |  |  |  |

**References**

1. Rahim Z, Mollers M, te Koppele-Vije A, de Beer J, Zaman K, Matin MA, et al. Characterization of Mycobacterium africanum subtype I among cows in a dairy farm in Bangladesh using spoligotyping. The Southeast Asian journal of tropical medicine and public health. 2007;38(4):706-13. Available from: <http://ovidsp.ovid.com/ovidweb.cgi?T=JS&PAGE=reference&D=med6&NEWS=N&AN=17883011>.

2. Islam MM, Siddiqui MAR, Haque MA, Baki MA, Majumder S, Parrish JJ, et al. Screening some major communicable diseases of AI bulls in Bangladesh. Livestock Research for Rural Development. 2007;19(6). Available from: <https://www.scopus.com/inward/record.uri?eid=2-s2.0-34249979736&partnerID=40&md5=5117555826183c0a4d5fef3321a49227>.

3. Rahim Z, Thapa J, Fukushima Y, van der Zanden AGM, Gordon SV, Suzuki Y, et al. Tuberculosis Caused by Mycobacterium orygis in Dairy Cattle and Captured Monkeys in Bangladesh: a New Scenario of Tuberculosis in South Asia. Transboundary and Emerging Diseases. 2017;64(6):1965-9.

4. Islam SKS, Rumi TB, Kabir SML, van der Zanden AGM, Kapur V, Rahman A, et al. Bovine tuberculosis prevalence and risk factors in selected districts of Bangladesh. PLoS One. 2020;15(11). Available from: 10.1371/journal.pone.0241717.

5. Islam MN, Khan MK, Khan MFR, Kostoulas P, Anisur Rahman AKM, Alam MM. Risk factors and true prevalence of bovine tuberculosis in Bangladesh. PLoS One. 2021;16(2 February). Available from: <https://www.scopus.com/inward/record.uri?eid=2-s2.0-85102160408&doi=10.1371%2fjournal.pone.0247838&partnerID=40&md5=198155c9591051aad1ca2f01884cc368>.

6. Sultana N, Pervin M, Sultana S, Mostaree M, Belal SMSH, Khan MAHNA. Pathological investigation and molecular detection of bacterial zoonotic diseases of slaughtered cattle in Bangladesh. Journal of Advanced Biotechnology and Experimental Therapeutics. 2022;5(2):257-68. Available from: <https://www.scopus.com/inward/record.uri?eid=2-s2.0-85129861685&doi=10.5455%2fjabet.2022.d113&partnerID=40&md5=3ed5fa1fcc49c49a17b04cced452fc17>.

7. Dravid MN, Joshi S, Bhardwaj RS, Khare PM. Differential identification of Mycobacterium tuberculosis from various clinical specimens from Sassoon General Hospital, Pune. Indian J Med Sci. 1992;46(2):43-5. Available from: <https://www.scopus.com/inward/record.uri?eid=2-s2.0-0026819778&partnerID=40&md5=aa736d823f69f930a2302b1646ec746e>.

8. Singh BB, Gumber S, Randhawa SS, Aradhana, Dhand NK. Prevalence of bovine tuberculosis and paratuberculosis in Punjab. Indian Veterinary Journal. 2004;81(11):1195-6. Available from: <https://www.scopus.com/inward/record.uri?eid=2-s2.0-9944220199&partnerID=40&md5=608ac6f8ce7177fbb5a9a50b2dd46d34>.

9. Parashar D, Srivastava RK, Chauhan DS, Sharma VD, Singh M, Lavania M, et al. Characterization of mycobacteria isolated from bovines by PRA-targetting hsp 65 gene region. Journal of Communicable Diseases. 2006;38(3):263-8. Available from: <https://www.scopus.com/inward/record.uri?eid=2-s2.0-33846542657&partnerID=40&md5=02bd00c4ddd7547e75dfa4cfe6361790>.

10. Rajkhowa S, Rajkhowa C, Hazarika GC. Prevalence of tuberculin reactors in mithuns (Bos frontalis) in India. Indian J Anim Sci. 2007;77(8):671-4. Available from: <https://www.scopus.com/inward/record.uri?eid=2-s2.0-38749131181&partnerID=40&md5=bcb1b9662b77d9a1f5ce7ca567b2afb0>.

11. Srivastava K, Chauhan DS, Gupta P, Singh HB, Sharma VD, Yadav VS, et al. Isolation of Mycobacterium bovis & M. tuberculosis from cattle of some farms in north India - Possible relevance in human health. Indian Journal of Medical Research. 2008;128(1):26-31. Available from: <https://www.scopus.com/inward/record.uri?eid=2-s2.0-55849091350&partnerID=40&md5=97c8f76a8f5e014f75ae9e9674b47b6f>.

12. Thakur A, Sharma M, Katoch VC, Dhar P, Katoch RC. A study on the prevalence of Bovine Tuberculosis in farmed dairy cattle in Himachal Pradesh. Vet World. 2010;3(9):409-14.

13. Kohli MD, Nambam B, Trivedi SS, Sherwal BL, Arora S, Jain A. PCR-based evaluation of tuberculous endometritis in infertile women of North India. Medical Journal of Reproduction and Infertility. 2011;12(1):9-14. Available from: <https://www.scopus.com/inward/record.uri?eid=2-s2.0-80053925872&partnerID=40&md5=c0f68c847d29f5c064ddfa447af0ef76>.

14. Thakur A, Sharma M, Katoch VC, Dhar P, Katoch RC. Detection of Mycobacterium bovis and Mycobacterium tuberculosis from Cattle: Possible Public Health Relevance. Indian J Microbiol. 2012;52(2):289-91.

15. Mittal M, Chakravarti S, Sharma V, Sanjeeth BS, Churamani CP, Kanwar NS. Evidence of Presence of Mycobacterium tuberculosis in Bovine Tissue Samples by Multiplex PCR: Possible Relevance to Reverse Zoonosis. Transboundary and Emerging Diseases. 2014;61(2):97-104.

16. Narnaware SD, Dahiya SS, Tuteja FC, Nagarajan G, Nath K, Patil NV. Pathology and diagnosis of Mycobacterium bovis in naturally infected dromedary camels (Camelus dromedarius) in India. Trop Anim Health Prod. 2015;47(8):1633-6.

17. Mukherjee F, Bahekar VS, Prasad A, Rana SK, Kanani A, Sharma GK, et al. Isolation of Mycobacterium tuberculosis from Antelope cervicapra and Gazelle bennettii in India and confirmation by molecular tests. EUROPEAN JOURNAL OF WILDLIFE RESEARCH. 2015;61(5):783-7.

18. Shukla SK, Chauhan A, Shukla S, Panigrahi M, Bhushan B, Sarvjeet SM, et al. Screening of Bovine Tuberculosis Cattle using the Tuberculin Skin Test in Barsana. J Pure Appl Microbiol. 2016;10(2):1527-32.

19. Shukla SK, Chauhan A, Shukla S, Panigrahi M, Bhushan B, Sarvjeet SM, et al. Detection and Identification of Bovine Tuberculosis in Indian Cattle (Bos indicus). J Pure Appl Microbiol. 2016;10(2):1579-84.

20. Bapat PR, Dodkey RS, Shekhawat SD, Husain AA, Nayak AR, Kawle AP, et al. Prevalence of zoonotic tuberculosis and associated risk factors in Central Indian populations. Journal of Epidemiology and Global Health. 2017;7(4):277-83. Available from: <https://www.scopus.com/inward/record.uri?eid=2-s2.0-85029232027&doi=10.1016%2fj.jegh.2017.08.007&partnerID=40&md5=d8fb77c855b3c2aa58541d316851aa28>.

21. Dabade G, Kale A, Athani BR, Sankri V, Pawar SL, Megeri SN. A study on zoonotic tuberculosis in selected rural areas of Bagalkot and Belgaum districts of Karnataka state. Journal of Clinical Tuberculosis and Other Mycobacterial Diseases. 2017;9:30-5. Available from: <https://www.scopus.com/inward/record.uri?eid=2-s2.0-85034625872&doi=10.1016%2fj.jctube.2017.10.002&partnerID=40&md5=1affc3616c5070804477bd7b5e7d815e>.

22. Sweetline Anne N, Ronald BSM, Kumar TMAS, Kannan P, Thangavelu A. Molecular identification of Mycobacterium tuberculosis in cattle. Veterinary Microbiology. 2017;198:81-7. Available from: <https://www.scopus.com/inward/record.uri?eid=2-s2.0-85006508033&doi=10.1016%2fj.vetmic.2016.12.013&partnerID=40&md5=cb545419f021f122ca9e99ad3b4c745d>.

23. Das R, Dandapat P, Chakrabarty A, Nanda PK, Bandyopadhyay S, Bandyopadhyay S. A cross-sectional study on prevalence of bovine tuberculosis in Indian and crossbred cattle in Gangetic delta region of West Bengal, India. International Journal of One Health. 2018;4:1-7. Available from: <https://www.scopus.com/inward/record.uri?eid=2-s2.0-85050389243&doi=10.14202%2fIJOH.2018.1-7&partnerID=40&md5=7830f8efb5bad71d85807660df34903c>.

24. Mukherjee F, Bahekar VS, Pasha SY, Kannan P, Prasad A, Rana SK, et al. Isolation and analysis of the molecular epidemiology and zoonotic significance of Mycobacterium tuberculosis in domestic and wildlife ruminants from three states in India. Rev Sci Tech. 2018;37(3):999-1012.

25. Srinivasan S, Easterling L, Rimal B, Niu XM, Conlan AJK, Dudas P, et al. Prevalence of Bovine Tuberculosis in India: A systematic review and meta-analysis. Transboundary and Emerging Diseases. 2018;65(6):1627-40.

26. Refaya AK, Kumar N, Raj D, Veerasamy M, Balaji S, Shanmugam S, et al. Whole-Genome Sequencing of a Mycobacterium orygis Strain Isolated from Cattle in Chennai, India. Microbiology Resource Announcements. 2019;8(40). Available from: 10.1128/MRA.01080-19.

27. Palaniyandi K, Kumar N, Veerasamy M, Kabir Refaya A, Dolla C, Balaji S, et al. Isolation and comparative genomics of Mycobacterium tuberculosis isolates from cattle and their attendants in South India. Scientific Reports. 2019;9(1). Available from: <https://www.scopus.com/inward/record.uri?eid=2-s2.0-85075784630&doi=10.1038%2fs41598-019-54268-x&partnerID=40&md5=3dad3854aafd7dc9536746d4512c5336>.

28. Duffy SC, Srinivasan S, Schilling MA, Stuber T, Danchuk SN, Michael JS, et al. Reconsidering Mycobacterium bovis as a proxy for zoonotic tuberculosis: a molecular epidemiological surveillance study. The Lancet Microbe. 2020;1(2):e66-e73. Available from: <https://www.scopus.com/inward/record.uri?eid=2-s2.0-85096335009&doi=10.1016%2fS2666-5247%2820%2930038-0&partnerID=40&md5=9daf2192824a6f1ec71286a06e7f928c>.

29. Bapat PR, Shekhawat SD, Husain AA, Dodkey RS, Daginawala HF, Singh LK, et al. Diagnostic Challenges and Prospects Associated With Zoonotic Tuberculosis of Central Nervous System. Basic and Clinical Neuroscience. 2020;11(5):619-30.

30. Rajhans U, Wankhede G, Ambore B, Chaudhari S, Nighot N, Dhaygude V, et al. Sero-diagnosis of Tuberculosis in Elephants in Maharashtra, India. J Threat Taxa. 2021;13(7):18713-8. Available from: <https://www.scopus.com/inward/record.uri?eid=2-s2.0-85109188870&doi=10.11609%2fjott.5502.13.7.18713-18718&partnerID=40&md5=3fd762fb90be314f345d6bc3c46b6a54>.

31. Refaya AK, Ramanujam H, Ramalingam M, Rao GVS, Ravikumar D, Sangamithrai D, et al. Tuberculosis caused by Mycobacterium orygis in wild ungulates in Chennai, South India. Transbound Emerg Dis. 2022;69(5):e3327-e33.

32. Jha VC, Morita Y, Dhakal M, Besnet B, Sato T, Nagai A, et al. Isolation of Mycobacterium spp. from milking buffaloes and cattle in Nepal. J Vet Med Sci. 2007;69(8):819-25.

33. Bhandari M, Thoen CO. Zoonotic tuberculosis in humans, elephants, and other animals in Nepal. In: Thoen CO, Steele JH, Kaneene JB, editors. Zoonotic Tuberculosis: Mycobacterium bovis and Other Pathogenic Mycobacteria: 3rd Edition: John Wiley & Sons, Inc.; 2014. p. 191-5.

34. Thapa J, Nakajima C, Maharjan B, Poudel A, Suzuki Y. Molecular characterization of Mycobacterium orygis isolates from wild animals of Nepal. Japanese Journal of Veterinary Research. 2015;63(3):151-8.

35. Thapa J, Paudel S, Sadaula A, Shah Y, Maharjan B, Kaufman GE, et al. Mycobacterium orygis-associated tuberculosis in free-ranging rhinoceros, Nepal, 2015. Emerging Infectious Diseases. 2016;22(3):570-2. Available from: <https://www.scopus.com/inward/record.uri?eid=2-s2.0-84958767371&doi=10.3201%2feid2203.151929&partnerID=40&md5=84cb14db60dac4fa3062cc1fca17661c>.

36. Gompo TR, Shrestha A, Ranjit E, Gautam B, Ale K, Shrestha S, et al. Risk factors of tuberculosis in human and its association with cattle TB in Nepal: A one health approach. One Health. 2020;10. Available from: 10.1016/j.onehlt.2020.100156.

37. Jayasumana MTLK, Dunuwila DMUNK, Palkumbura PGAS, Mudalige R, Dharmawardana IVP, Wijesundera RRMKK, et al. Detection of Mycobacterium bovis in cattle lungs from two abattoirs in Western and North central provinces of Sri Lanka. Journal of the National Science Foundation of Sri Lanka. 2021;49(1):99-109. Available from: <https://www.scopus.com/inward/record.uri?eid=2-s2.0-85109050474&doi=10.4038%2fjnsfsr.v49i1.9873&partnerID=40&md5=9cfbe8fdb03c8eabb39d949797b7e9fb>.

38. Kanameda M, Ekgatat M. Isolation of mycobacterium bovis from the water buffalo (bubalus bubalis). Trop Anim Health Prod. 1995;27(4):227-8. Available from: <https://www.scopus.com/inward/record.uri?eid=2-s2.0-0029396282&doi=10.1007%2fBF02250697&partnerID=40&md5=e3c7185a20a8f66f5db258280c16f2dc>.

39. Kaewamatawong T, Banlunara W, Rungsipipat A, Pirarat N, Puranaveja S, Sommanustweechai A. Disseminated Tuberculosis in Captive Malayan Tapir (Tapirus indicus). Thai Journal of Veterinary Medicine. 2010;40(4):427-31.

40. Songthammanuphap S, Puthong S, Pongma C, Buakeaw A, Prammananan T, Warit S, et al. Detection of Mycobacterium tuberculosis complex infection in Asian elephants (Elephas maximus) using an interferon gamma release assay in a captive elephant herd. SCIENTIFIC REPORTS. 2020;10(1). Available from: 10.1038/s41598-020-71099-3.

41. Singhla T, Boonyayatra S. Prevalence, Risk Factors, and Diagnostic Efficacy of Bovine Tuberculosis in Slaughtered Animals at the Chiang Mai Municipal Abattoir, Thailand. Frontiers in Veterinary Science. 2022;9. Available from: 10.3389/fvets.2022.846423.

42. Singhla T, Pikulkaew S, Boonyayatra S. Performance of Loop-Mediated Isothermal Amplification Technique in Milk Samples for the Diagnosis of Bovine Tuberculosis in Dairy Cattle Using a Bayesian Approach. Pathogens. 2022;11(5). Available from: 10.3390/pathogens11050573.

43. Rogers RJ, Donald BA, Schultz K. The distribution of Mycobacterium bovis in Queensland cattle herds with observations on the laboratory diagnosis of tuberculosis. Australian veterinary journal. 1980;56(11):542-6. Available from: <http://ovidsp.ovid.com/ovidweb.cgi?T=JS&PAGE=reference&D=med2&NEWS=N&AN=7018487>.

44. Corner LA, Barrett RH, Lepper AW, Lewis V, Pearson CW. A survey of mycobacteriosis of feral pigs in the Northern Territory. Australian veterinary journal. 1981;57(12):537-42. Available from: <http://ovidsp.ovid.com/ovidweb.cgi?T=JS&PAGE=reference&D=med2&NEWS=N&AN=7041875>.

45. Hein WR, Tomasovic AA. An abattoir survey of tuberculosis in feral buffaloes. Australian veterinary journal. 1981;57(12):543-7. Available from: <http://ovidsp.ovid.com/ovidweb.cgi?T=JS&PAGE=reference&D=med2&NEWS=N&AN=7041876>.

46. Robinson RC, Phillips PH, Stevens G, Storm PA. An outbreak of Mycobacterium bovis infection in fallow deer (Dama dama). Aust Vet J. 1989;66(7):195-7.

47. Pang SC, Clayton AS, Harrison RH. Culture-positive tuberculosis in Western Australia. Aust N Z J Med. 1992;22(2):109-13.

48. Thompson PJ, Cousins DV, Gow BL, Collins DM, Williamson BH, Dagnia HT. Seals, seal trainers, and Mycobacterial infection. American Review of Respiratory Disease. 1993;147(1):164-7.

49. Cousins DV, Francis BR, Casey R, Mayberry C. Mycobacterium bovis infection in a goat. Australian Veterinary Journal, 70: 262-263. 1993;70:262-3.

50. Cousins DV, Williams SN, Reuter R, Forshaw D, Chadwick B, Coughran D, et al. Tuberculosis in wild seals and characterisation of the seal bacillus. Aust Vet J. 1993;70(3):92-7.

51. McInerney J, Small K, Caley P. Prevalence of Mycobacterium bovis infection in feral pigs in the Northern Territory. Aust Vet J 1995 :448-51. 1995;72(12):448-51.

52. Cousins DV, Dawson DJ. Tuberculosis due to Mycobacterium bovis in the Australian population: cases recorded during 1970-1994. International Journal of Tuberculosis and Lung Disease. 1999;3(8):715-21.

53. Lumb R, Bastian I, Dawson D, Gilpin C, Havekort F, Howard P, et al. Tuberculosis in Australia: bacteriologically confirmed cases and drug resistance, 2000: report of the Australian Mycobacterium Laboratory Reference Network. Commun Dis Intell Q Rep. 2002;26(2):226-33.

54. Lumb R, Bastian I, Dawson D, Gilpin C, Haverkort F, James G, et al. Tuberculosis in Australia: bacteriologically confirmed cases and drug resistance, 2001. Commun Dis Intell Q Rep. 2003;27(2):173-80.

55. Lumb R, Bastian I, Gilpin C, Jelfs P, Keehner T, Sievers A. Tuberculosis in Australia: Bacteriologically confirmed cases and drug resistance, 2005 A report of the Australian Mycobacterium Reference Laboratory Network. Communicable Diseases Intelligence. 2007;31(1):80-6.

56. Lumb R, Bastian I, Gilpin C, Jelfs P, Keehner T, Sievers A. Tuberculosis in Australia: Bacteriologically confirmed cases and drug resistance, 2006 a report of the australian mycobacterium reference laboratory network. Communicable Diseases Intelligence. 2008;32(1):12-7.

57. Lumb R, Bastion I, Carter R, Jelfs P, Keehner T, Sievers A. Tuberculosis in Australia: bacteriologically confirmed cases and drug resistance, 2007. A report of the Australian Mycobacterium Reference Laboratory Network. Commun Dis Intell Q Rep. 2009;33(3):298-303.

58. Ingram PR, Bremner P, Inglis TJ, Murray RJ, Cousins DV. Zoonotic tuberculosis: On the decline. Communicable Diseases Intelligence. 2010;34(3):339-41.

59. Sergeant E, Happold J, Langstaff I. Evaluation of Australian surveillance for freedom from bovine tuberculosis. Aust Vet J. 2017;95(12):474-9.

60. Sergeant ESG, Happold J, Langstaff I, Hutchison J, Aeema. Evaluation of Australian surveillance for freedom from bovine tuberculosis. Epidemiologie et Sante Animale, No 59-602011. p. 325-7.

61. Zhao D, Wu CD, Ning Z. Chapter 23. Bovine Tuberculosis in China. In: Thoen CO, Steele JH, Gilsdorf MJ, editors. Mycobacterium bovis Infection in Animals and Humans. Iowa, USA: Blackwell Publishing Professional; 2006.

62. Chen Y, Chao Y, Deng Q, Liu T, Xiang J, Chen J, et al. Potential challenges to the Stop TB Plan for humans in China; cattle maintain M. bovis and M. tuberculosis. Tuberculosis (Edinb). 2009;89(1):95-100.

63. Hu XD, Jia K, Zhang GH, Lin ZX, Xia HY, Zhang DW, et al. The Use of a Mixture of esat-6 and cfp-10 Protein as Antigens in ELISA for the Diagnosis of Bovine Tuberculosis in South China. J Anim Vet Adv. 2011;10(14):1863-6.

64. Sun Z, Cao R, Tian M, Zhang X, Zhang X, Li Y, et al. Evaluation of Spoligotyping and MIRU-VNTR for Mycobacterium bovis in Xinjiang, China. Res Vet Sci. 2012;92(2):236-9.

65. Han Z, Gao J, Shahzad M, Meng X, Liu M, Zhang K, et al. Seroprevalence of bovine tuberculosis infection in yaks (Bos grunniens) on the Qinghai-Tibetan Plateau of China. Trop Anim Health Prod. 2013;45(6):1277-9.

66. Muller B, Durr S, Alonso S, Hattendorf J, Laisse CJM, Parsons SDC, et al. Zoonotic Mycobacterium bovis-induced tuberculosis in humans. Emerging infectious diseases. 2013;19(6):899-908. Available from: <http://ovidsp.ovid.com/ovidweb.cgi?T=JS&PAGE=reference&D=med10&NEWS=N&AN=23735540>.

67. Yang L, Wang C, Wang H, Meng Q, Wang Q. Evaluation of MIRU-VNTR for typing of Mycobacterium bovis isolated from Sika deer in Northeast China. BMC Vet Res. 2015;11:93.

68. Ji LY, Xu DL, Yin SP, Liu HC, Li GL, Jiang Y, et al. First Report in China on the Identification and Drug Sensitivity of Mycobacterium elephantis Isolated from the Milk of a Cow with Mastitis. Biomed Environ Sci. 2017;30(7):501-7.

69. Liu YH, Kang Q, Yang B, Li F, Li XQ, Zhang L, et al. Prevalence of bovine tuberculosis in the Aksu Region of Xinjiang, China, between 1985 and 2016. Arq Bras Med Vet Zootec. 2019;71 (02). Available from: 10.1590/1678-4162-10550.

70. Hajiaheman Y, Yang Y, Shayilanbieke N, Jin G. Mycobacterium culturing and drug resistance of osteoarticular tuberculosis in Xinjiang, China. Medicine (Baltimore). 2020;99(16):e19697.

71. Xu F, Tian L, Li Y, Zhang X, Qi Y, Jing Z, et al. High prevalence of extrapulmonary tuberculosis in dairy farms: Evidence for possible gastrointestinal transmission. PLoS One. 2021;16(3):e0249341.

72. Song Y-H, Li D, Zhou Y, Zhao B, Li J-M, Shi K, et al. Prevalence of Bovine Tuberculosis in Yaks Between 1982 and 2020 in Mainland China: A Systematic Review and Meta-Analysis. Vector borne and zoonotic diseases (Larchmont, NY). 2021;21(6):397-405. Available from: <http://ovidsp.ovid.com/ovidweb.cgi?T=JS&PAGE=reference&D=med20&NEWS=N&AN=33646056>.

73. Gong QL, Chen Y, Tian T, Wen X, Li D, Song YH, et al. Prevalence of bovine tuberculosis in dairy cattle in China during 2010-2019: A systematic review and meta-analysis. PLoS Negl Trop Dis. 2021;15(6):e0009502.

74. Borja E, Borja LF, Prasad R, Tunabuna T, Toribio J-ALML. A Retrospective Study on Bovine Tuberculosis in Cattle on Fiji: Study Findings and Stakeholder Responses. Frontiers in veterinary science. 2018;5:270. Available from: <http://ovidsp.ovid.com/ovidweb.cgi?T=JS&PAGE=reference&D=pmnm4&NEWS=N&AN=30417003>.

75. Urabe K, Hasimoto C. The Incidence in Human Tuberculosis of the bovine Type of Tubercle Bacilli in Japan. Kekkaku. 1941;19(10):695-701.

76. Une Y, Mori T. Tuberculosis as a zoonosis from a veterinary perspective. Comparative Immunology Microbiology And Infectious Diseases. 2007;30(5-6):415-25.

77. Shimao T. Control of cattle TB in Japan. Kekkaku. 2010;85(8):661-6.

78. Vongxay K, Conlan JV, Khounsy S, Dorny P, Fenwick S, Thompson RC, et al. Seroprevalence of major bovine-associated zoonotic infectious diseases in the Lao People's Democratic Republic. Vector Borne Zoonotic Dis. 2012;12(10):861-6.

79. Fakhruzzaman MNN, Abidin N, Aziz ZA, Lim WF, Richard JJ, Noorliza MN, et al. Diversified lineages and drug-resistance profiles of clinical isolates of Mycobacterium tuberculosis complex in Malaysia. International Journal of Mycobacteriology. 2019;8(4):320-8.

80. Lekko YM, Che-Amat A, Ooi PT, Omar S, Mohd-Hamdan DT, Linazah LS, et al. Detection of Mycobacterium tuberculosis complex antibodies in free-ranged wild boar and wild macaques in selected districts in Selangor and reevaluation of tuberculosis serodetection in captive Asian elephants in Pahang, Peninsular Malaysia. The Journal of veterinary medical science. 2021;83(11):1702-7. Available from: <http://ovidsp.ovid.com/ovidweb.cgi?T=JS&PAGE=reference&D=med19&NEWS=N&AN=34544936>.

81. de Lisle GW, Havill PF. Mycobacteria isolated from deer in New Zealand from 1970-1983. N Z Vet J. 1985;33(8):138-40.

82. Collins DM, De Lisle GW, Gabric DM. Geographic distribution of restriction types of Mycobacterium bovis isolates from brush-tailed possums (Trichosurus vulpecula) in New Zealand. J Hyg (Lond). 1986;96(3):431-8.

83. de Lisle GW, Collins DM, Loveday AS, Young WA, Julian AF. A report of tuberculosis in cats in New Zealand, and the examination of strains of Mycobacterium bovis by DNA restriction endonuclease analysis. N Z Vet J. 1990;38(1):10-3.

84. Coleman JD, Jackson R, Cooke MM, Grueber L. Prevalence and spatial distribution of bovine tuberculosis in brushtail possums on a forest-scrub margin. N Z Vet J. 1994;42(4):128-32.

85. Lugton IW, Johnstone AC, Morris RS. Mycobacterium bovis infection in New Zealand hedgehogs (Erinaceus europaeus). New Zealand Veterinary Journal. 1995;43(7):342-5.

86. Pfeiffer DU, Hickling GJ, Morris RS, Patterson KP, Ryan TJ, Crews KB. The epidemiology of Mycobacterium bovis infection in brushtail possums (Trichosurus vulpecula Kerr) in the Hauhungaroa Ranges, New Zealand. N Z Vet J. 1995;43(7):272-80.

87. Lugton IW, Wobeser G, Morris RS, Caley P. Epidemiology of Mycobacterium bovis infection in feral ferrets (Mustela furo) in New Zealand: II. Routes of infection and excretion. N Z Vet J. 1997;45(4):151-7.

88. Lugton IW, Wilson PR, Morris RS, Nugent G. Epidemiology and pathogenesis of Mycobacterium bovis infection of red deer (Cervus elaphus) in New Zealand. NEW ZEALAND VETERINARY JOURNAL. 1998;46(4):147-56.

89. Gay G, Burbidge HM, Bennett P, Fenwick SG, Dupont C, Murray A, et al. Pulmonary Mycobacterium bovis infection in a dog. NEW ZEALAND VETERINARY JOURNAL. 2000;48(3):78-81.

90. Cooke MM, Gear AJ, Naidoo A, Collins DM. Accidental Mycobacterium bovis infection in a veterinarian. NEW ZEALAND VETERINARY JOURNAL. 2002;50(1):36-8.

91. Corner LA, Norton S. Resolution of Mycobacterium bovis infection in wild brushtail possums (Trichosurus vulpecula). N Z Vet J. 2003;51(1):40-2.

92. Mackintosh CG, de Lisle GW, Collins DM, Griffin JF. Mycobacterial diseases of deer. N Z Vet J. 2004;52(4):163-74.

93. Griffin JF, Chinn DN, Rodgers CR. Diagnostic strategies and outcomes on three New Zealand deer farms with severe outbreaks of bovine tuberculosis. Tuberculosis (Edinb). 2004;84(5):293-302.

94. Coleman JD, Coleman MC, Warburton B. Trends in the incidence of tuberculosis in possums and livestock, associated with differing control intensities applied to possum populations. New Zealand veterinary journal. 2006;54(2):52-60. Available from: <http://ovidsp.ovid.com/ovidweb.cgi?T=JS&PAGE=reference&D=med6&NEWS=N&AN=16596155>.

95. de Lisle GW, Yates GF, Caley P, Corboy RJ. Surveillance of wildlife for Mycobacterium bovis infection using culture of pooled tissue samples from ferrets (Mustela furo). New Zealand Veterinary Journal. 2005;53(1):14-8.

96. de Lisle GW, Kawakami RP, Yates GF, Collins DM. Isolation of Mycobacterium bovis and other mycobacterial species from ferrets and stoats. Veterinary microbiology. 2008;132(3-4):402-7. Available from: <http://ovidsp.ovid.com/ovidweb.cgi?T=JS&PAGE=reference&D=med7&NEWS=N&AN=18632227>.

97. Dawson KL, Bell A, Kawakami RP, Coley K, Yates G, Collins DM. Transmission of Mycobacterium orygis (M. tuberculosis complex species) from a tuberculosis patient to a dairy cow in New Zealand. J Clin Microbiol. 2012;50(9):3136-8.

98. de Lisle GW, Yates GF, Coleman JD. Isolation of Mycobacterium bovis from brushtail possums with non-visible lesions. New Zealand Veterinary Journal. 2009;57(4):221-4.

99. Sinclair JA, Dawson KL, Buddle BM. The effectiveness of parallel gamma-interferon testing in New Zealand's bovine tuberculosis eradication programme. Prev Vet Med. 2016;127:94-9.

100. Price-Carter M, Brauning R, de Lisle GW, Livingstone P, Neill M, Sinclair J, et al. Whole Genome Sequencing for Determining the Source of Mycobacterium bovis Infections in Livestock Herds and Wildlife in New Zealand. Frontiers in Veterinary Science. 2018;5. Available from: 10.3389/fvets.2018.00272.

101. Baker MG, Lopez LD, Cannon MC, De Lisle GW, Collins DM. Continuing Mycobacterium bovis transmission from animals to humans in New Zealand. Epidemiology and Infection. 2006;134(5):1068-73.

102. Roe WD, Lenting B, Kokosinska A, Hunter S, Duignan PJ, Gartrell B, et al. Pathology and molecular epidemiology of Mycobacterium pinnipedii tuberculosis in native New Zealand marine mammals. PLoS One. 2019;14(2). Available from: 10.1371/journal.pone.0212363.

103. Reis AC, Ramos B, Pereira AC, Cunha MV. The hard numbers of tuberculosis epidemiology in wildlife: A meta-regression and systematic review. Transboundary and Emerging Diseases. 2021;68(6):3257-76.

104. Wee S-H, Kim C-H, More SJ, Nam HM. Mycobacterium bovis in Korea: an update. Veterinary journal (London, England : 1997). 2010;185(3):347-50. Available from: <http://ovidsp.ovid.com/ovidweb.cgi?T=JS&PAGE=reference&D=med8&NEWS=N&AN=19713136>.

105. Jeon B-Y, Kim S-C, Je S, Kwak J, Cho J-E, Woo J-T, et al. Evaluation of enzyme-linked immunosorbent assay using milk samples as a potential screening test of bovine tuberculosis of dairy cows in Korea. Res Vet Sci. 2010;88(3):390-3. Available from: <http://ovidsp.ovid.com/ovidweb.cgi?T=JS&PAGE=reference&D=med8&NEWS=N&AN=20060551>.

106. Lee H, Kim J-M, Jang Y, Lee K, Baek K, Lee B, et al. Bovine tuberculosis in an Asian small-clawed otter (Aonyx cinerea) in the Republic of Korea. Journal of veterinary diagnostic investigation : official publication of the American Association of Veterinary Laboratory Diagnosticians, Inc. 2015;27(5):651-5. Available from: <http://ovidsp.ovid.com/ovidweb.cgi?T=JS&PAGE=reference&D=med12&NEWS=N&AN=26289719>.

107. Je S, Ku BK, Jeon B-Y, Kim J-M, Jung S-C, Cho S-N. Extent of Mycobacterium bovis transmission among animals of dairy and beef cattle and deer farms in South Korea determined by variable-number tandem repeats typing. Veterinary microbiology. 2015;176(3-4):274-81. Available from: <http://ovidsp.ovid.com/ovidweb.cgi?T=JS&PAGE=reference&D=med12&NEWS=N&AN=25676210>.

108. Ku BK, Jeon B-Y, Kim JM, Jang Y-B, Jang Y, Yu SY, et al. Mycobacterium bovis infection in a wild sow (Sus scrofa): first case in Korea. Journal of veterinary science. 2016;17(3):427-9. Available from: <http://ovidsp.ovid.com/ovidweb.cgi?T=JS&PAGE=reference&D=med13&NEWS=N&AN=26726026>.

109. Kim BJ, Kim GN, Kim BR, Jeon CO, Jeong J, Lee SH, et al. Description of mycobacterium chelonae subsp. Bovis subsp. nov., isolated from cattle (bos taurus coreanae), emended description of mycobacterium chelonae and creation of mycobacterium chelonae subsp. chelonae subsp. nov. Int J Syst Evol Microbiol. 2017;67(10):3882-7.

110. Kim N, Jang Y, Kim JK, Ryoo S, Kwon KH, Kim M, et al. Molecular and genomic features of Mycobacterium bovis strain 1595 isolated from Korean cattle. Journal of veterinary science. 2017;18(S1):333-41. Available from: <http://ovidsp.ovid.com/ovidweb.cgi?T=JS&PAGE=reference&D=med14&NEWS=N&AN=28385004>.

111. Jang Y, Ryoo S, Lee H, Kim N, Lee H, Park S-Y, et al. Isolation of Mycobacterium bovis from Free-Ranging Wildlife in South Korea. J Wildl Dis. 2017;53(1):181-5. Available from: <http://ovidsp.ovid.com/ovidweb.cgi?T=JS&PAGE=reference&D=med14&NEWS=N&AN=27809648>.

112. Ku BK, Jeon B-Y, Kim JM, Jang Y-B, Lee H, Choi JY, et al. Investigation of bovine tuberculosis outbreaks by using a trace-back system and molecular typing in Korean Hanwoo beef cattle. Journal of veterinary science. 2018;19(1):45-50. Available from: <http://ovidsp.ovid.com/ovidweb.cgi?T=JS&PAGE=reference&D=med15&NEWS=N&AN=28693300>.

113. Roh SG, Jang YH, Kim J, Lee K, So B, Choi EJ. A rare case of bovine tuberculosis caused by Mycobacterium bovis in a domestic rabbit. Korean Journal of Veterinary Research. 2020;60(2):85-8.

114. Kim TW, Jang YH, Jeong MK, Seo Y, Park CH, Kang S, et al. Single-nucleotide polymorphism-based epidemiological analysis of Korean Mycobacterium bovis isolates. Journal of veterinary science. 2021;22(2):e24. Available from: <http://ovidsp.ovid.com/ovidweb.cgi?T=JS&PAGE=reference&D=med19&NEWS=N&AN=33774940>.
